# Induction agents for emergency tracheal intubation in critically ill adults: a systematic review and network meta-analysis

**DOI:** 10.1186/s13054-026-06067-w

**Published:** 2026-05-12

**Authors:** Fernando G. Zampieri, Raysa C. Schmidt, Bruno A. M. P. Besen, Fernando J. D. S. Ramos, François Lamontagne, Neill K. J. Adhikari, Flávio G. R. Freitas, Flávia R. Machado

**Affiliations:** 1 Department of Critical Care Medicine, University of Alberta, CSB 2-124, 8440 112 St NW, Edmonton, AB T6G 2B7 Canada; 2 Brazilian Research in Intensive Care Network (BRICNet), São Paulo, Brazil; 3 Intensive Care Department, Hospital São Paulo, Escola Paulista de Medicina, Universidade Federal de São Paulo, São Paulo, SP Brazil; 4 Department of Medicine, Université de Sherbrooke, Sherbrooke, Canada; 5 Department of Critical Care Medicine, Sunnybrook Health Sciences Centre, University of Toronto, Toronto, Canada

**Keywords:** Intubation, Rapid sequence induction, Etomidate, Ketamine, Propofol, Network meta-analysis, Critically ill

## Abstract

**Background:**

Etomidate, ketamine, and propofol are all used as induction agents for emergency tracheal intubation in critically ill adults but it remains uncertain which agent should be preferable.

**Methods:**

We searched MEDLINE and Embase (inception to December 2025) for randomized controlled trials comparing etomidate, ketamine, propofol, or ketamine–propofol combination (ketofol) for emergency or rapid sequence intubation in critically ill adults. We performed random-effects network meta-analysis using the frequentist framework. The primary outcome was short-term mortality (28–30 day, or ICU/in-hospital mortality when unavailable). Secondary outcomes included cardiovascular collapse, post-induction hypotension, vasopressor use, first-pass intubation success, and peri-intubation cardiac arrest. Certainty of evidence was assessed using the CINeMA framework.

**Results:**

Nine trials (4,672 patients, four treatments) were included. Ketamine and etomidate probably result in similar mortality (OR 0.96, 95% CI 0.80–1.16; ${I}^{2}$ = 30%; moderate certainty). Evidence for other mortality comparisons was very uncertain: ketamine vs propofol (OR 1.53, 0.80–2.93; 1 trial; low certainty) and etomidate vs propofol (OR 0.63, 0.32–1.24; indirect only; very low certainty). Compared with etomidate, ketamine probably increases cardiovascular collapse (OR 1.44, 1.20–1.71; moderate certainty) and may increase post-induction hypotension (OR 1.34, 1.07–1.68; low certainty) and peri-intubation vasopressor use (OR 1.45, 1.21–1.74; low certainty). There was probably little or no difference in first-pass intubation success or cardiac arrest.

**Conclusions:**

Etomidate and ketamine probably result in similar mortality, but confidence intervals are compatible with clinically important differences in either direction—ketamine probably causes more peri-intubation hemodynamic instability. Beyond one trial, no randomized evidence exists for propofol in emergency intubation of critically ill adults.

**Supplementary Information:**

The online version contains supplementary material available at 10.1186/s13054-026-06067-w.

## Introduction

Emergency tracheal intubation in critically ill patients is a high-risk procedure. In the INTUBE study, an international cohort of nearly 3,000 patients, major adverse peri-intubation events occurred in 45% of cases, including cardiovascular instability in 43% and cardiac arrest in 3% [1]. Peri-intubation cardiac arrest is independently associated with increased mortality, with hemodynamic instability at induction identified as a key risk factor [2]. The choice of induction agent is one of the few modifiable factors that may influence peri-intubation hemodynamics.

The choice of induction agent varies globally. In the INTUBE cohort spanning 29 countries, propofol was the most frequently used induction agent for emergency intubation, whereas etomidate, ketamine, and midazolam were each used less often [1]. Practice differs in North America, where etomidate accounts for approximately 90% of rapid sequence intubations in emergency departments, with ketamine use growing but still below 15% [3, 4]. Etomidate provides reliable hemodynamic stability but inhibits 11 $\beta$-hydroxylase, causing transient adrenal suppression even after a single dose—a concern particularly in septic patients [5]. Ketamine exerts sympathomimetic effects through endogenous catecholamine release, but may cause myocardial depression in catecholamine-depleted patients [6]. Propofol causes dose-dependent vasodilation that limits its use in hemodynamically unstable patients, yet it remains the most widely used induction agent for emergency intubation worldwide. Ketamine–propofol combinations (ketofol) have been proposed to balance these pharmacodynamic profiles. 

Several pairwise meta-analyses have compared etomidate and ketamine for emergency intubation [7–10], generally finding no statistically significant difference in mortality; a Bayesian meta-analysis, however, found a moderate probability that ketamine may reduce mortality [11]. These analyses were limited to the etomidate–ketamine comparison and could not incorporate evidence from trials involving propofol or ketofol. Two recent trials have substantially changed the evidence landscape: the RSI trial [12] (2,359 patients), the largest randomized comparison of etomidate and ketamine to date; and the PROMINE trial [13], the first randomized trial comparing propofol with ketamine for emergency intubation in critically ill patients, thereby enabling a network meta-analysis beyond the etomidate–ketamine dyad.

We therefore conducted a systematic review and network meta-analysis comparing etomidate, ketamine, propofol, and ketofol for emergency tracheal intubation in critically ill adults, with short-term mortality as the primary outcome. 

## Methods

This systematic review and network meta-analysis was registered on PROSPERO (CRD420251251225) and is reported in accordance with the PRISMA-NMA extension [14].

### Eligibility criteria

We included parallel-group randomized controlled trials comparing etomidate, ketamine (including esketamine or S-ketamine), propofol, or ketamine–propofol combination (ketofol) as induction agents for emergency or rapid sequence intubation in critically ill or acutely ill adults ($\ge$ 16 years). Studies were eligible regardless of clinical setting (emergency department, intensive care unit, prehospital, or acute hospital ward). Co-interventions (opioids, neuromuscular blocking agents, preoxygenation strategies) were permitted if applied similarly across treatment arms. 

We excluded observational studies, crossover trials, simulation or volunteer studies, conference abstracts without sufficient data for risk of bias assessment and effect estimation, studies of elective or operating-room intubations, procedural sedation without intubation, studies focused on post-intubation sedation rather than induction, pediatric studies in which adult data could not be isolated, elective surgical populations without acute critical illness, and studies where the randomized intervention was not the hypnotic induction agent. Only English-language publications were included; one eligible study published in Turkish (Cinar 2011 [15]) was retained because sufficient information was available from its English-language abstract and AI-assisted translation (Claude, Anthropic).

### Information sources and search strategy

We searched MEDLINE (via PubMed) and Embase (via Ovid) from inception to December 2025. Forward citation searching of included trials was performed to identify additional relevant studies. Trial registries (ClinicalTrials.gov, WHO ICTRP) were consulted for contextual information. The complete search strategies are provided in the Supplement.

### Study selection and data extraction

Search results were imported into a systematic review platform (Rayyan, Rayyan Systems Inc., Cambridge, MA) for deduplication and screening. Two reviewers independently screened titles and abstracts; disagreements at this stage were resolved by consensus. This was followed by full-text assessment of potentially eligible reports against the predefined criteria. Disagreements were resolved through discussion. Reasons for exclusion at the full-text stage were recorded. Data were extracted independently by two reviewers using a standardized form and verified against source publications. Extracted information included study characteristics (authors, year, country, setting, single vs multicenter, funding), population (sample size, age, sex, severity scores, baseline hemodynamics, vasopressor use), interventions (agent, dose, co-induction agents, neuromuscular blocker type and dose), and arm-level outcome data with definitions and time windows.

### Risk of bias assessment

Risk of bias was assessed using the Cochrane Risk of Bias 2 (RoB 2) tool across five domains [16]: randomization process, deviations from intended interventions, missing outcome data, measurement of the outcome, and selection of the reported result. Each study was rated as low risk, some concerns, or high risk of bias.

### Outcomes

The primary outcome was short-term mortality, defined as 28–30 day mortality where reported, or ICU or in-hospital mortality when 28–30 day data were unavailable. Secondary outcomes included: cardiovascular collapse (composite of hypotension, vasopressor initiation or escalation, and/or cardiac arrest, as defined by individual studies); post-induction hypotension (blood pressure below study-defined thresholds within 0–15 min); new or escalated vasopressor use (analyzed at peri-intubation and 24-h time points separately); first-pass intubation success; and peri-intubation cardiac arrest. All binary outcomes were analyzed as odds ratios (OR) with 95% confidence intervals (CI).

### Statistical analysis

Arm-level binary data were converted to study-level contrasts (log OR and standard error) using the pairwise() function from the *meta* package (version 8.2—1) [17]. Random-effects network meta-analysis was performed using the frequentist approach implemented in the *netmeta* package (version 3.3—1) in R (version 4.5.2) [18]. Etomidate was designated as the reference treatment. Esketamine and S-ketamine were grouped under the ketamine node.

Between-study heterogeneity was quantified using ${I}^{2}$ and ${\tau }^{2}$. Global inconsistency was evaluated using the design-by-treatment interaction test, and local inconsistency was assessed through node-splitting. Treatment rankings were summarized using P-scores [19].

Pairwise meta-analysis of etomidate versus ketamine was performed using the Paule–Mandel estimator for ${\tau }^{2}$ with 95% prediction intervals.

### Subgroup and sensitivity analyses

Pre-specified subgroup analyses for the primary outcome included stratification by clinical setting (emergency department, ICU, or mixed—studies enrolling across both ED and ICU settings were classified as mixed because patients could not be disaggregated by location) and overall risk of bias. A planned subgroup analysis by baseline vasopressor use was not feasible because only three of nine studies reported this information. Similarly, degree of RSI protocolization, a predefined effect modifier, could not be formally evaluated because the distinction between strict and pragmatic protocols was not sufficiently categorical across studies. Other clinically important subgroups—such as patients with heart failure or by primary diagnosis—could not be examined because individual-study data were not available at this level.

Sensitivity analyses excluded: (a) studies where the ketamine arm included a midazolam adjunct (Cinar 2011 [15], Punt 2014 [20]); and (b) analyses collapsing or removing ketofol as a separate treatment node.

### Certainty of evidence

Certainty of evidence for key comparisons was assessed using the CINeMA framework (Confidence in Network Meta-Analysis) [21], evaluating six domains: within-study bias, reporting bias, indirectness, imprecision, heterogeneity, and incoherence. Ratings were classified as high, moderate, low, or very low certainty. Because the etomidate–ketamine comparison was informed entirely by direct evidence (7 trials), certainty for this comparison was assessed without indirect contributions. The etomidate–propofol comparison relied entirely on indirect evidence through the ketamine node.

### Use of large language models

This review made extensive use of large language models (LLMs). The search strategy was designed with assistance from Gemini 3.0 Pro (Google). Screening was performed using Rayyan.ai, and selected full-text articles were retrieved by the first and last authors. Data extraction was performed by Claude Opus 4.6 (Anthropic) from source PDFs, with each extraction independently verified against the source publication by at least two investigators (F.J.D.S.R., F.R.M., and the first author). Risk of bias assessments were drafted by the LLM and independently reviewed by F.R.M. CINeMA certainty-of-evidence assessments were performed by F.G.Z. and reviewed by F.R.M. Statistical analysis code (R) was written with assistance from Claude Opus 4.6 and verified by the first author. In all cases, LLM outputs were treated as preliminary drafts requiring human verification—no extracted data point, risk-of-bias judgement, or analytical decision was accepted without investigator review. Notably, the near-identical summary statistics between Agarwal 2025 and Srivilaithon 2023 (detailed in the Supplement) were first identified by the LLM during data extraction, not by the human reviewers—illustrating a potential advantage of LLM-assisted workflows in detecting data anomalies across large volumes of extracted information. A complete numerical audit verified all extracted values against source PDFs and confirmed reproducibility of all analytical outputs. While formal validation studies comparing LLM-assisted extraction with traditional dual-reviewer approaches in critical care systematic reviews are still lacking, this workflow ensured that the final dataset reflects human-verified values throughout.

## Results

### Study selection

**Fig. 1:**
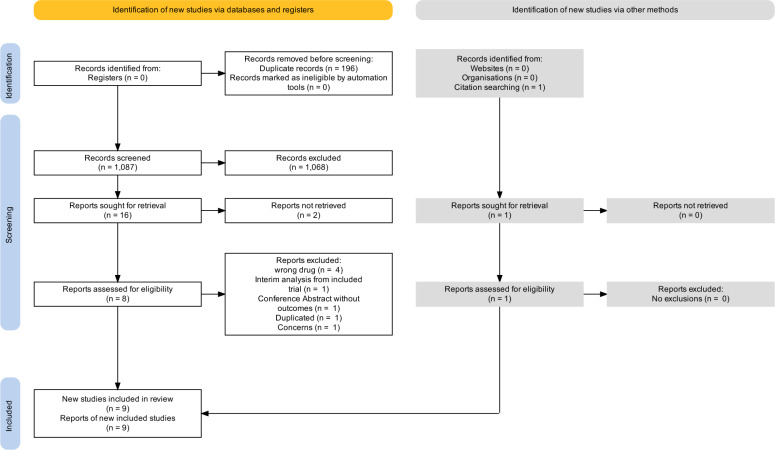
PRISMA flow diagram. Study identification, screening, and inclusion process. MEDLINE and Embase were searched from inception to December 2025. Nine randomized controlled trials met the inclusion criteria

The search identified 1,087 records after deduplication. After title/abstract screening and full-text review, 9 trials met the inclusion criteria (Fig. 1). One additional trial (Agarwal 2025 [22]) initially met the eligibility criteria but was excluded after independent review identified that its published summary statistics—including means, standard deviations, medians, and interquartile ranges across all baseline variables—were near-identical to those of Srivilaithon 2023 [23], a previously included study conducted in a different country with a different sample size (India vs Thailand; $N$ = 80 vs $N$ = 260). Srivilaithon 2023 was retained as the original publication: it was published two years earlier (2023 vs 2025), enrolled a larger sample, appeared in a higher-impact peer-reviewed journal, and had trial registry documentation (TCTR20210213001), whereas Agarwal 2025 reported no trial registration. A detailed comparison is provided in the Supplement.

### Study characteristics

The 9 included trials [12, 13, 15, 23–27] enrolled 4,672 patients across four treatment nodes: etomidate (8 trials), ketamine (8 trials), propofol (1 trial), and ketofol (1 trial). Studies were conducted in ICU (5 trials), emergency department (2 trials), and mixed settings (2 trials), across six countries. Sample sizes ranged from 22 to 2,359 patients. Baseline severity varied across studies but was measured using different scales (SAPS II, APACHE II, APACHE III, SAPS 3, qSOFA), precluding direct comparison; per-study values are reported in the Supplement. Baseline hemodynamics also varied: mean systolic blood pressure ranged from approximately 113 mmHg (Srivilaithon 2023, sepsis-only [23]) to 139 mmHg (Knack 2023 [27]), and the proportion of patients receiving vasopressors at enrollment ranged from 22 to 37% among the three studies reporting this variable. The characteristics of included studies are presented in Table 1.

**Table 1:** Characteristics of included studies

Study	N	Population	Comparison	Induction dose	NMBA‖	Primary outcome (original)	Key secondary outcomes
Jabre 2009 (KETASED) [24]	469	Prehospital/ED, multicenter (77), France	E vs K	E 0.3 vs K 2 mg/kg	Sux 1 mg/kg	Maximum SOFA (days 1–3)	28—d mortality; cortisol levels
Cinar 2011* [15]	22	ICU, single-center, Turkey	E vs K	E 0.3 vs K 2 mg/kg + midaz 0.03 mg/kg	None	Heart rate, MAP	Cortisol levels; SOFA day 9
Punt 2014* [20]	301	ICU, single-center, Netherlands	E vs SK	E 0.2–0.3 vs SK 0.5 mg/kg + midaz 2.5 mg	Roc	28—d mortality	Cortisol levels; ICU LOS
Smischney 2019 (KEEP PACE) [25]	152	ICU, single-center, USA	E vs Ketofol	E 0.15 vs KP 0.5 + 0.5 mg/kg†	Sux (57%) / Roc (36%)	MAP change at 5 min	New vasopressor use; cortisol levels
Matchett 2022 (EvK) [26]	791	ICU, single-center, USA	E vs K	E 0.2–0.3 vs K 1–2 mg/kg	Roc (81%) / Sux (18%)	Day 7 survival	28—d mortality
Knack 2023 [27]	143	ED, single-center, USA	E vs K	E 0.3 vs K 2 mg/kg	Sux (92%)	Maximum SOFA‡	30—d mortality; hypotension
Srivilaithon 2023 [23]	260	ED (sepsis), single-center, Thailand	E vs K	E 0.2–0.3 vs K 1–2 mg/kg	Sux 1.5 mg/kg§	28—d survival	Hypotension; vasopressor use 24 h
Casey 2025 (RSI) [12]	2,359	ED + ICU, multicenter (14), USA	E vs K	E 0.2–0.3 vs K 1–2 mg/kg	Roc (69%) / Sux (31%)	28—d in-hospital mortality	CV collapse
Schmidt 2025 (PROMINE) [13]	175	ICU, multicenter (2), Brazil	P vs ESK	P 1.5 vs ESK 2 mg/kg	Roc 1.2 mg/kg (98%)	Lowest MAP within 10 min	Hospital mortality; CV collapse

N: patients analyzed, E: etomidate, K: ketamine, SK: S-ketamine, ESK: esketamine, P: propofol, KP: ketamine–propofol admixture, Sux: succinylcholine, Roc: rocuronium, ED: emergency department, ICU: intensive care unit, NMBA: neuromuscular blocking agent, CV: cardiovascular, LOS: length of stay, MAP: mean arterial pressure, SOFA: Sequential Organ Failure Assessment

^*^Ketamine arm included midazolam adjunct — flagged for sensitivity analysis

^†^Both drugs at reduced doses; fentanyl 50 µg co-administered

^‡^Primary outcome changed mid-trial from mortality to maximum SOFA

^§^NMBA use differed significantly between groups

‖NMBA reporting varies across studies: some report intended treatment, others actual administration

### Risk of bias

**Fig. 2:**
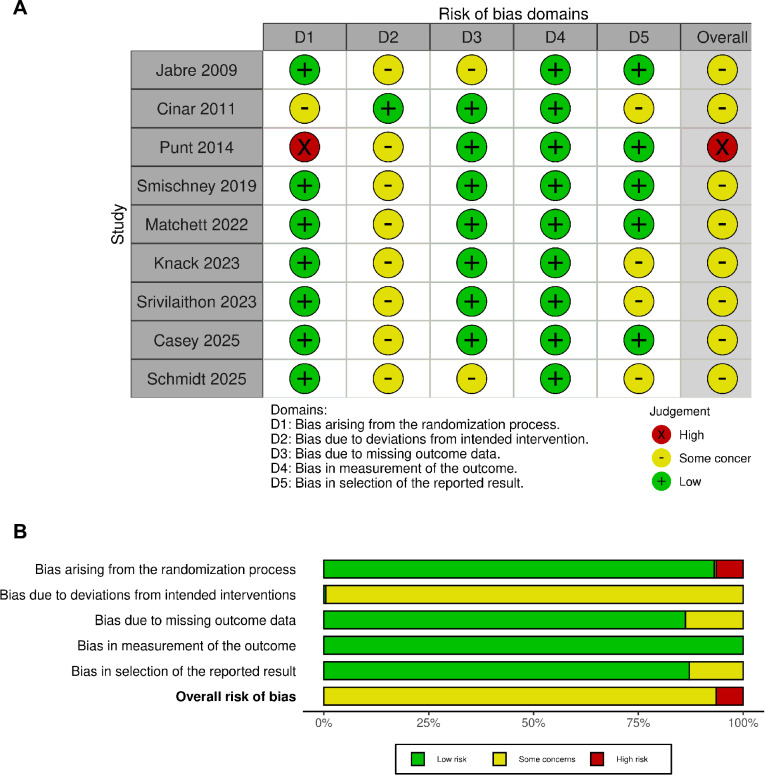
Risk of bias assessment (Cochrane RoB 2 tool). (**A**) Traffic-light plot showing domain-level judgements for each study. D1 = randomization process; D2 = deviations from intended interventions; D3 = missing outcome data; D4 = measurement of the outcome; D5 = selection of the reported result. (**B**) Summary plot showing the distribution of risk-of-bias judgements across all studies. Eight studies were rated as “some concerns” and one (Punt 2014) as “high risk,” driven by the cluster-randomized design and midazolam adjunct in the ketamine arm

Eight studies were rated as having “some concerns” and one (Punt 2014 [20]) as “high risk” of bias (Fig. 2). No trial blinded the clinician performing intubation except Cinar 2011 (identical syringes prepared by an uninvolved clinician [15]). Five trials blinded outcome assessors, ICU staff, or adjudicators. Mortality, as an objective outcome, was considered less susceptible to bias from lack of blinding.

### Primary outcome: short-term mortality

**Fig. 3:**
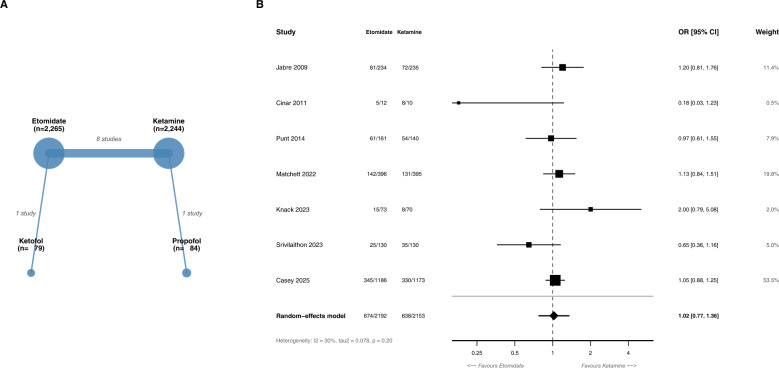
Network geometry and pairwise meta-analysis of etomidate vs ketamine for short-term mortality. (**A**) Network geometry for the mortality analysis. Node size is proportional to the total number of patients randomized to each treatment. Edge thickness is proportional to the number of studies for each direct comparison. The etomidate–ketamine comparison dominates the network (7 of 9 studies). (**B**) Forest plot showing the pairwise random-effects meta-analysis for etomidate vs ketamine (7 studies, 4,345 patients). Squares represent individual study estimates (size proportional to weight); the diamond represents the pooled estimate. The pooled OR was 1.02 (95% CI 0.77–1.36; 95% prediction interval 0.47–2.21; I^2^ = 30%), consistent with no difference in mortality between agents

Nine trials contributed to the mortality network meta-analysis (Fig. 3A). Network heterogeneity was low (${I}^{2}$ = 30%, ${\tau }^{2}$ = 0.017). Formal assessment of global inconsistency was not possible because only one comparison (etomidate vs ketamine) was informed by multiple studies; node-splitting similarly could not be performed as no comparison had both direct and indirect evidence.

Ketamine and etomidate probably result in similar short-term mortality (NMA estimate: OR 0.96, 95% CI 0.80–1.16; 7 direct studies, 4,345 patients; moderate certainty) (Fig. 3B, Table 2). In the direct pairwise meta-analysis using the Paule–Mandel estimator, the pooled OR was 1.02 (95% CI 0.77–1.36; 95% prediction interval 0.47–2.21), consistent with the NMA result; the small numerical difference reflects the different ${\tau }^{2}$ estimators and the network structure. The comparison of ketamine vs propofol was based on a single trial and showed that ketamine may increase mortality compared with propofol, although the evidence is very uncertain (OR 1.53, 95% CI 0.80–2.93; low certainty). The indirect estimate for etomidate vs propofol was very uncertain (OR 0.63, 95% CI 0.32–1.24; very low certainty). Ketamine and ketofol may result in similar mortality (OR 0.84, 95% CI 0.41–1.72; 1 trial; low certainty). P-scores for mortality ranked propofol first (0.84), followed by ketofol (0.54), ketamine (0.38), and etomidate (0.24); however, these rankings should be interpreted with extreme caution given the low to very low certainty of evidence for all comparisons except etomidate–ketamine.

**Table 2:** Summary of network meta-analysis results: primary and secondary outcomes

Outcome / comparison	Studies, k (N)	Evidence	OR (95% CI)	I^2^	Certaintyᵈ
Short-term mortality (primary outcome)					
Ketamine vs etomidate	7 (4,345)	Direct	0.96 (0.80–1.16)	30%	Moderate
Ketofol vs etomidate	1 (152)	Direct	0.84 (0.41–1.72)	-	Low
Propofol vs etomidate	0 (—)	Indirect	0.63 (0.32–1.24)	-	Very low
Ketamine vs Propofol	1 (175)	Direct	1.53 (0.80–2.93)	-	Low
Secondary outcomes — Ketamine vs etomidateᵇ					
Cardiovascular collapse	3 [2] (3,331)	NMA	1.44 (1.20–1.71)	0%	Moderate
Post-induction hypotension	4 [3] (2,871)	NMA	1.34 (1.07–1.68)	0%	Low
Vasopressor, peri-intubation	5 [3] (3,413)	NMA	1.45 (1.21–1.74)	0%	Low
Vasopressor, 24 hoursᵃ	2 [2] (2,421)	PW	0.51 (0.16–1.56)	93%	Very low
First-pass intubation success	5 [4] (3,718)	NMA	0.95 (0.77–1.16)	0%	Moderate
Peri-intubation cardiac arrest	7 [5] (4,142)	NMA	1.13 (0.70–1.82)	0%	Low

OR: odds ratio, CI: confidence interval, NMA: network meta-analysis, PW: pairwise meta-analysis. For mortality, I^2^ refers to network-level heterogeneity; for secondary outcomes, I^2^ is comparison-specific. For mortality comparisons, OR < 1 favors the first-named treatment (lower mortality). For secondary outcomes, OR > 1 indicates a higher event rate with ketamine vs etomidate. Certainty of evidence assessed using the CINeMA framework

ᵃSubstantial heterogeneity; individual study ORs were 1.15 (Casey 2025) and 3.63 (Srivilaithon 2023) in the etomidate vs ketamine direction

ᵇStudies, k [direct]: total studies contributing to the NMA [direct ketamine vs etomidate comparisons]. Remaining studies contribute indirect evidence through the propofol or ketofol nodes

ᶜSecondary outcomes for propofol comparisons are available in the Supplement (eFigures)

ᵈReasons for downgrading certainty: K vs E mortality: imprecision; Ketofol vs E: imprecision, indirectness; P vs E: bias, indirectness, imprecision; K vs P: imprecision, indirectness; CV collapse: bias; hypotension: bias, indirectness; vasopressor (peri): bias, indirectness; vasopressor (24 h): bias, heterogeneity, imprecision; first-pass: bias; cardiac arrest: bias, imprecision. See eTables in Supplement for detailed CINeMA assessments

All results are summarized in Table [tab:results].

### Secondary outcomes

Compared with etomidate, ketamine probably increases cardiovascular collapse (OR 1.44, 95% CI 1.20–1.71; moderate certainty) and may increase post-induction hypotension (OR 1.34, 95% CI 1.07–1.68; low certainty) and peri-intubation vasopressor use (OR 1.45, 95% CI 1.21–1.74; low certainty). Vasopressor use at 24 h showed substantial heterogeneity (${I}^{2}$ = 93%) and was not reliably estimable. No study reported vasopressor use at a 1-h time point, precluding analysis at that protocol-specified interval. 

There is probably little or no difference in first-pass intubation success (OR 0.95, 95% CI 0.77–1.16; moderate certainty). Ketamine may result in little or no difference in peri-intubation cardiac arrest (OR 1.13, 95% CI 0.70–1.82; low certainty).

### Subgroup and sensitivity analyses

The effect of etomidate versus ketamine on mortality was consistent across clinical settings (ED, ICU, mixed; test for subgroup differences $p$ = 0.83) and across risk-of-bias categories ($p$ = 0.88). Excluding studies with midazolam adjuncts did not materially change the results. Collapsing ketofol into the ketamine node (OR 0.95, 0.80–1.12) or removing the ketofol node entirely (OR 0.96, 0.80–1.16) yielded similar estimates. A post-hoc sensitivity analysis excluding the largest trial (Casey 2025, which contributed $\sim$ 50% of all patients) showed that cardiovascular collapse (OR 1.59, 1.12–2.24) and peri-intubation vasopressor use (OR 1.50, 1.06–2.15) remained significantly higher with ketamine, whereas the hypotension finding was attenuated (OR 1.01, 0.59–1.73). For vasopressor use at 24 h, excluding Casey resolved the heterogeneity (${I}^{2}$ dropped from 93%), with the single remaining study (a sepsis-only population) showing higher 24-h vasopressor use with etomidate. Cardiac arrest was unchanged (OR 1.09, 0.60–1.95). eFigures in the Supplement.

### Certainty of evidence

Certainty was moderate for the etomidate–ketamine mortality comparison (downgraded for imprecision: the confidence interval does not exclude a clinically important absolute difference of 2–3%) and for cardiovascular collapse and first-pass success. Certainty was low or very low for all other comparisons and outcomes, primarily due to imprecision, indirectness (especially for comparisons involving propofol, which relied on a single trial or indirect evidence), and heterogeneous outcome definitions (Table [tab:results]).

## Discussion

In this systematic review and network meta-analysis of 9 randomized trials enrolling 4,672 critically ill adults, etomidate and ketamine probably result in similar short-term mortality, but confidence intervals do not exclude clinically important differences. Ketamine probably causes more peri-intubation cardiovascular instability than etomidate. Evidence for propofol and ketofol was limited to single trials.

Our mortality findings are consistent with all five recent pairwise meta-analyses comparing etomidate and ketamine [7–10]. This analysis adds propofol and ketofol to the comparison for the first time. Notably, a large observational study from the Brazilian Airway Registry (BARCO) reported higher 28-day mortality with etomidate than with ketamine (60.5% vs 54.4%) [28]. The discordance between that observational finding and the randomized evidence synthesized here likely reflects residual confounding—etomidate may be preferentially selected for patients perceived to be at higher hemodynamic risk—though the possibility of a real but modest effect that randomized trials have been underpowered to detect individually cannot be excluded.

Ketamine was associated with higher odds of cardiovascular collapse, post-induction hypotension, and peri-intubation vasopressor use compared with etomidate. This may seem paradoxical given ketamine’s sympathomimetic properties, but critically ill patients are often catecholamine-depleted, blunting the indirect sympathetic stimulation on which ketamine’s hemodynamic stability depends. In this setting, ketamine’s direct myocardial depressant effect may predominate. The protocol specified vasopressor analysis at up to 30 min, 1 h, and 24 h; at the 24-h time point, substantial heterogeneity (${I}^{2}$ = 93%) precluded reliable estimation, highlighting the lack of standardized hemodynamic outcome definitions in this field.

Two recent trials deserve specific discussion. The RSI trial enrolled 2,359 patients—nearly half of all patients in this review—across 14 sites in the United States and was the first with mortality as the primary endpoint (28-day mortality 28.1% ketamine vs 29.1% etomidate) [12]. It also provided the most detailed hemodynamic data: cardiovascular collapse occurred in 22.1% of ketamine vs 17.0% of etomidate patients, largely driven by vasopressor escalation. These patterns are consistent with the pooled estimates in our network meta-analysis. Sensitivity analysis excluding the RSI trial showed that the cardiovascular collapse and peri-intubation vasopressor findings persisted, whereas the hypotension finding was attenuated. Induction doses varied across trials (Table 1): protocol-specified ketamine doses ranged from 1–2 mg/kg to a fixed 2 mg/kg, but actual administered doses were often lower (median 1.1 mg/kg in Matchett 2022, 1.6 mg/kg in Casey 2025). These trial doses may not fully reflect real-world practice, where many clinicians substantially reduce induction doses in hemodynamically unstable patients. Whether dose differences contributed to the heterogeneity in hemodynamic effects warrants further investigation, and future trials should consider protocolized dose adjustment based on hemodynamic status. PROMINE is the only randomized trial comparing propofol with ketamine in critically ill adults [13]. The trial enrolled 175 ICU patients in Brazil using propofol 1.5 mg/kg and esketamine 2 mg/kg; results showed that esketamine was associated with higher blood pressure in the periintubation period although numeric mortality was higher with ketamine than propofol (60% vs 50%, OR 1.53, 95% CI 0.80–2.93). The propofol dose used (1.5 mg/kg) is higher than the 0.5–1.0 mg/kg that many clinicians use in hemodynamically unstable patients, and the hemodynamic findings from PROMINE should be interpreted in this context. Because PROMINE is the sole trial connecting propofol to the network, all propofol comparisons depend on it. The indirect estimate for etomidate versus propofol (OR 0.63, 95% CI 0.32–1.24; very low certainty) suggests large statistical imprecision remains for the comparison between propofol and etomidate. Similarly, the direct ketamine–propofol comparison from PROMINE alone is too imprecise to draw definitive conclusions: the confidence interval is compatible with propofol reducing mortality by 20% or increasing it nearly threefold. What these estimates do show is that propofol is not obviously worse than etomidate or ketamine for mortality, which should motivate larger trials. Propofol has several theoretical advantages that support equipoise: it is the most widely used induction agent for emergency intubation worldwide, does not cause adrenal suppression, and its dose-dependent hemodynamic effects may be attenuated at lower doses (0.5–1.0 mg/kg) than the 1.5 mg/kg used in PROMINE. Moreover, propofol-related hemodynamic instability may be amenable to prevention through protocolized co-interventions such as preemptive vasopressor administration. Notably, a secondary analysis of the INTUBE cohort found that propofol was associated with peri-intubation cardiovascular instability (OR 1.28, 95% CI 1.05–1.57) but not with life-threatening cardiovascular collapse [29], suggesting that propofol-related hemodynamic effects may be transient and amenable to management. No trial has directly compared propofol with etomidate. The single ketofol trial (KEEP PACE [25]) used reduced doses of both components with fentanyl co-administration, limiting its generalizability.

Drug selection for emergency intubation is one of several modifiable factors that may influence peri-intubation outcomes in critically ill patients. Other components of a peri-intubation bundle—including standardized checklists, hemodynamic monitoring with an arterial line, fluid loading, and preemptive vasopressor administration—may mitigate cardiovascular collapse regardless of the induction agent chosen, and their interaction with drug selection warrants prospective evaluation. The best available evidence compares ketamine with etomidate: mortality is probably similar but confidence intervals do not exclude clinically important differences, and ketamine probably causes more peri-intubation hemodynamic instability. For propofol, PROMINE is the first and only randomized trial in this setting; its results are hypothesis-generating but insufficient to guide practice. No trial has compared propofol with etomidate. Further trials—particularly testing propofol against etomidate and ketamine—are needed. Standardized definitions for hemodynamic outcomes, particularly vasopressor use, are also needed to allow meaningful comparison across future studies.

This review has several limitations. First, most included trials were open-label, which may introduce performance bias for subjectively assessed outcomes such as hypotension. However, mortality—our primary outcome—is objective and less susceptible to ascertainment bias. Second, the treatment network is sparse: propofol and ketofol each connect to the network through a single trial, and the etomidate–propofol comparison is entirely indirect. Formal consistency testing was not informative because no comparison had both direct and indirect evidence. Third, definitions of hemodynamic outcomes varied considerably across studies, particularly for hypotension thresholds and vasopressor time windows, contributing to clinical heterogeneity. Indeed, vasopressor use at 24 h showed an ${I}^{2}$ of 93%, precluding reliable estimation. Fourth, a planned subgroup analysis by baseline vasopressor use was not feasible because only three studies reported this variable, despite its likely role as an effect modifier. Fifth, we included two studies (Cinar 2011 [15], Punt 2014 [20]) in which the ketamine arm received a midazolam adjunct; sensitivity analyses excluding these trials yielded consistent results. Punt 2014 used a cluster-crossover design (alternating treatment periods [20]); we used the study-reported estimates, which may not fully account for the cluster structure. Finally, our search was restricted to English-language publications, although one Turkish-language study was included based on its English abstract and AI-assisted translation.

## Conclusions

Etomidate and ketamine probably result in similar short-term mortality, but confidence intervals do not exclude clinically important differences in either direction, and ketamine is probably associated with more peri-intubation hemodynamic instability. Evidence for propofol is limited to a single trial. Large uncertainty remains regarding the optimal drug for emergency intubation of critically ill patients. Further trials are needed.

## Supplementary Information

Additional file 1.

## Data Availability

No datasets were generated or analysed during the current study.
