# Supplementary material for: Induction agents for emergency tracheal intubation in critically ill adults: a systematic review and network meta-analysis
Source: Crit Care. 2026 May 12;30:296. doi: 10.1186/s13054-026-06067-w (PMC13245039; doi:10.1186/s13054-026-06067-w)
Supplement: Supplementary file 1 — Additional file 1. [file 13054_2026_6067_MOESM1_ESM.pdf]

Electronic Supplementary Material

Induction agents for emergency tracheal intubation in critically ill adults: a systematic review and network meta-analysis

Zampieri FG, Schmidt RC, Besen BAMP, Ramos FJDS, Lamontagne F, Adhikari NKJ, Freitas FGR, Machado FR

Contents

1 Search Strategies **3**
1.1 MEDLINE (via PubMed) . . . 3
1.2 Embase (via Ovid) . . . 3
1.3 Forward citation searching . . . 4
1.4 Trial registry screening . . . 4

2 PRISMA Extension for Network Meta-Analysis Checklist **5**

3 Supplementary Figures **7**

4 Certainty of Evidence (CINeMA Framework) **19**
4.1 Primary outcome: short-term mortality . . . 19
4.2 Key secondary outcomes (Ketamine vs Etomidate) . . . 21

5 Data Extractions **22**
5.1 Summary of mortality data . . . 22
5.2 Per-study extraction forms . . . 23
5.2.1 Study 1: Jabre 2009 (KETASED) . . . 23
5.2.2 Study 2: Cinar 2011 . . . 24
5.2.3 Study 3: Punt 2014 . . . 25
5.2.4 Study 4: Smischney 2019 (KEEP PACE) . . . 26
5.2.5 Study 5: Matchett 2022 (EvK) . . . 28
5.2.6 Study 6: Knack 2023 . . . 29
5.2.7 Study 7: Srivilaithon 2023 . . . 30
5.2.8 Study 8: Casey 2025 (RSI Trial) . . . 31
5.2.9 Study 9: Schmidt 2025 (PROMINE) . . . 33
5.3 Risk of bias summary across studies . . . 36

6 Expression of Concern: Possible Data Duplication (Agarwal 2025) 37
6.1 Papers under comparison . . . 37
6.2 Context . . . 37
6.3 Baseline characteristics . . . 38
6.4 Within-arm baseline values . . . 39
6.5 Intubation conditions . . . 39
6.6 Outcomes . . . 40
6.7 Note on p-values . . . 40
6.8 Summary . . . 40

7 Study Protocol 41

1 Search Strategies

Searches were conducted from database inception through December 2025. No language restrictions were applied in the search; however, only English-language publications were included at the screening stage, with one exception: a Turkish-language study (Cinar 2011) was retained because sufficient information was available from its English abstract and AI-assisted translation.

1.1 MEDLINE (via PubMed)

Search executed on December 10, 2025.

```
(
  "Intubation, Intratracheal"[Mesh] OR intubat*[tiab] OR
  "tracheal intubation"[tiab] OR "rapid sequence"[tiab] OR
  "rapid-sequence"[tiab] OR RSI[tiab]
)
AND
(
  "Emergency Service, Hospital"[Mesh] OR
  "Intensive Care Units"[Mesh] OR
  emergency[tiab] OR "emergency department"[tiab] OR ED[tiab] OR
  "critical illness"[Mesh] OR "critically ill"[tiab] OR
  ICU[tiab] OR "prehospital"[tiab]
)
AND
(
  etomidate[tiab] OR ketamine[tiab] OR propofol[tiab] OR
  amidate[tiab] OR diprivan[tiab] OR ketofol[tiab]
)
AND
(
  randomized controlled trial[pt] OR controlled clinical trial[pt] OR
  random*[tiab] OR trial[tiab] OR "clinical trial"[pt]
)
NOT
(
  animals[mh] NOT humans[mh]
)
```

1.2 Embase (via Ovid)

Search executed on December 10, 2025.

```
1.  exp endotracheal intubation/ or exp rapid sequence induction/
2.  (intubat* or "tracheal intubation" or "rapid sequence"
    or "rapid-sequence" or RSI).ti,ab.
3.  1 or 2
4.  exp emergency ward/ or exp intensive care unit/
    or exp critical illness/
5.  (emergency or "emergency department" or ED
    or "critically ill" or ICU or prehospital).ti,ab.
6.  4 or 5
7.  (etomidate or ketamine or propofol or amidate
    or diprivan or ketofol).ti,ab.
8.  exp etomidate/ or exp ketamine/ or exp propofol/
9.  7 or 8
10. 3 and 6 and 9
11. randomized controlled trial/ or controlled clinical trial/
12. (random* or trial or RCT).ti,ab.
13. 11 or 12
14. 10 and 13
15. (animal/ or nonhuman/) not human/
16. 14 not 15
```

1.3 Forward citation searching

Forward citation searching was performed on all included trials using Google Scholar. Reference lists of recent systematic reviews (Kotani 2023, Koroki 2024, Daghmouri 2025, de Morais 2025) were also screened. No additional eligible studies were identified beyond those captured by the database searches.

1.4 Trial registry screening

ClinicalTrials.gov and WHO ICTRP were screened for completed or ongoing trials comparing eligible induction agents for emergency intubation. One registered trial (NCT05092152, PROMINE) was identified and included based on manuscript data. No additional eligible completed trials were identified.

2 PRISMA Extension for Network Meta-Analysis Checklist

The following checklist is based on the PRISMA extension statement for systematic reviews incorporating network meta-analyses (Hutton et al., *Ann Intern Med* 2015;162:777–784). Items marked with "S" are NMA-specific additions.

Section/Topic	#	Checklist Item	Reported In
Title			
Title	1	Identify the report as a systematic review incorporating a network meta-analysis	Title
Abstract			
Structured summary	2	Provide a structured summary	Abstract
Introduction			
Rationale	3	Describe the rationale for the review, including why a network meta-analysis was conducted	Introduction, para 3
Objectives	4	Explicit statement of questions with reference to PICOS	Introduction, para 4
Methods			
Protocol & registration	5	Indicate existence of protocol and registration	Methods, para 1
Eligibility criteria	6	Specify study characteristics and eligible treatments in the network	Eligibility criteria
Information sources	7	Describe all information sources	Information sources
Search	8	Full electronic search strategy for at least one database	ESM, Search Strategies
Study selection	9	State the process for selecting studies	Study selection
Data collection	10	Describe data extraction methods and confirmation processes	Study selection
Data items	11	List and define all variables sought	Outcomes; ESM data extractions
Geometry of the network	S1	Describe methods to explore network geometry and potential biases	Statistical analysis; Figure 3A
Risk of bias in studies	12	Describe methods for assessing risk of bias	Risk of bias assessment
Summary measures	13	State principal summary measures; describe treatment rankings	Statistical analysis
Planned methods of analysis	14	Methods of combining results, handling multi-arm trials, variance structure	Statistical analysis
Inconsistency	S2	Statistical methods to evaluate consistency of direct and indirect evidence	Statistical analysis

Continued on next page

Section/Topic	#	Checklist Item	Reported In
Risk of bias across studies	15	Assessment of cumulative evidence bias	Certainty of evidence
Additional analyses	16	Methods of additional analyses, indicating pre-specified	Subgroup and sensitivity
Results			
Study selection	17	Numbers screened, assessed, included, with flow diagram	Study selection; Figure 1
Network structure	S3	Network graph for visualization	Figure 3A
Network geometry summary	S4	Overview of network characteristics, gaps, potential biases	Study characteristics; Discussion
Study characteristics	18	Characteristics and citations for each study	Table 1; ESM extractions
Risk of bias in studies	19	Risk of bias for each study	Figure 2; ESM eFigures
Individual study results	20	Summary data for each treatment group, effect estimates	ESM data extractions
Synthesis of results	21	Results of each meta-analysis with confidence intervals; league tables or rankings	Results; Table 2; eFigures
Inconsistency	S5	Results from investigations of inconsistency	Primary outcome
Risk of bias across studies	22	Results of bias assessment across studies	Certainty of evidence; ESM
Additional analyses	23	Results of sensitivity or subgroup analyses	Subgroup and sensitivity; ESM eFigures
Discussion			
Summary of evidence	24	Main findings including strength of evidence	Discussion, paras 1–3
Limitations	25	Limitations at study, outcome, and review level	Discussion, para 5
Conclusions	26	General interpretation and implications for future research	Conclusions
Funding			
Funding	27	Sources of funding and role of funders	Title page

3 Supplementary Figures

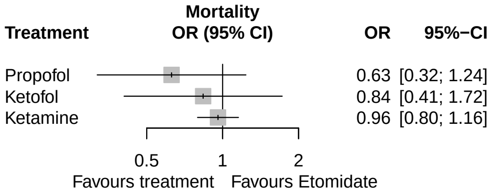

eFigure e1. Network meta-analysis forest plot for short-term mortality. All treatments compared with etomidate (reference). Random-effects model. OR < 1 favors the comparator treatment (lower mortality).

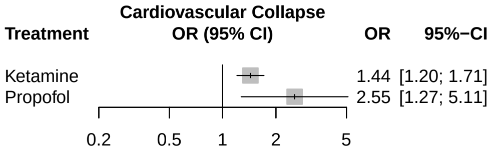

eFigure e2. Forest plot for cardiovascular collapse. Three studies contributed to a three-node NMA (etomidate, ketamine, propofol). OR > 1 indicates higher event rate vs etomidate.

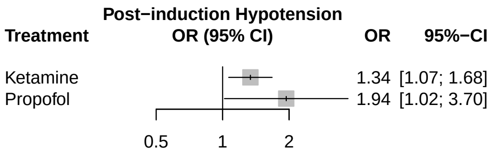

eFigure e3. Forest plot for post-induction hypotension. Four studies contributed; definitions varied across studies (SBP <80, SBP <90, or MAP <65 mmHg).

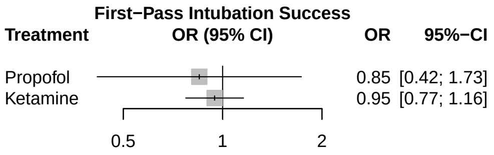

eFigure e4. Forest plot for first-pass intubation success. Five studies contributed. OR < 1 indicates lower first-pass success vs etomidate.

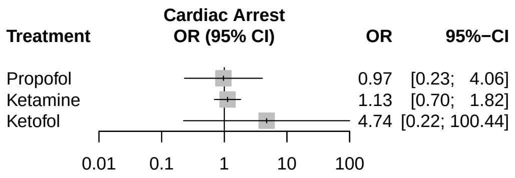

eFigure e5. Forest plot for peri-intubation cardiac arrest. Seven studies contributed. Event rates were low across all studies.

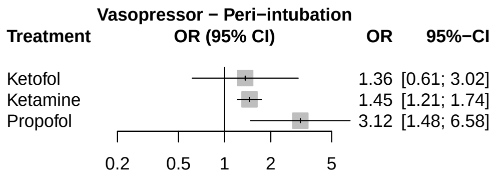

eFigure e6. Forest plot for vasopressor use, peri-intubation (induction to 2–5 minutes). Five studies contributed.

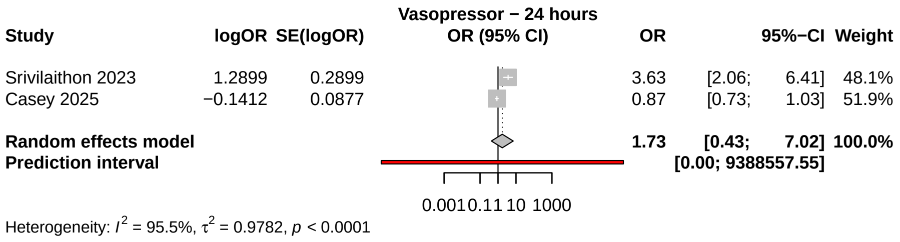

eFigure e7. Forest plot for vasopressor use at 24 hours (etomidate vs ketamine direction; OR > 1 indicates higher rate with etomidate). Two studies contributed. Substantial heterogeneity (I^2 = 93%) precludes reliable pooled estimation. Individual study ORs were 1.15 (Casey 2025) and 3.63 (Srivilaithon 2023). In Table 2, this result is presented in the ketamine vs etomidate direction (OR 0.51, 95% CI 0.16–1.56).

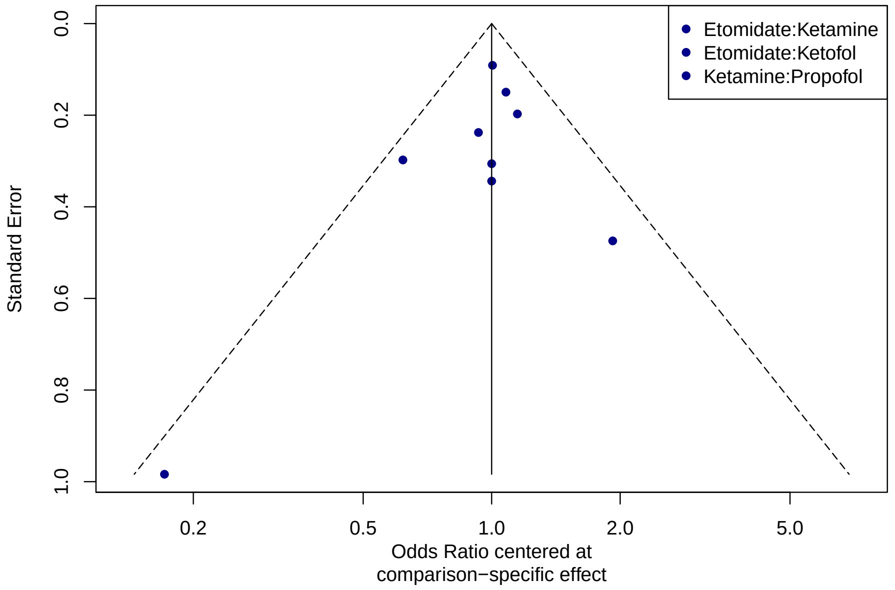

eFigure e8. Comparison-adjusted funnel plot for the mortality network meta-analysis. No clear asymmetry was observed, although interpretation is limited by the small number of studies.

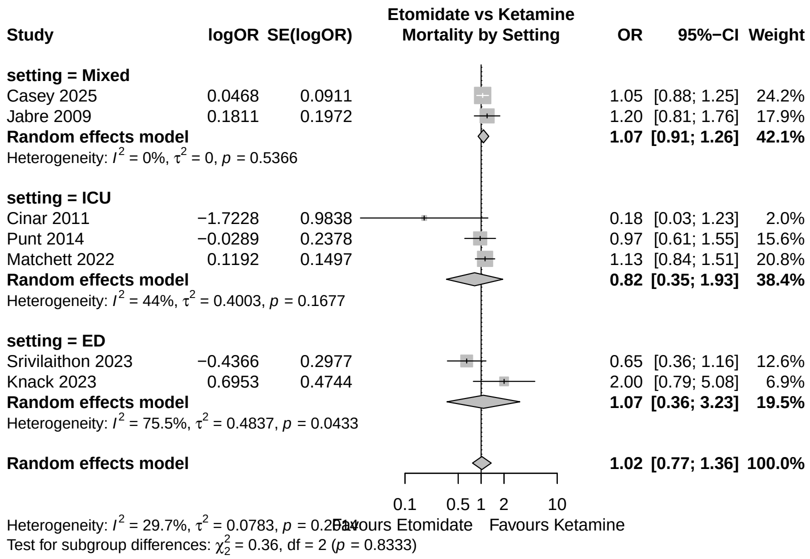

eFigure e9. Subgroup analysis for etomidate vs ketamine mortality by clinical setting (ED, ICU, mixed). Test for subgroup differences: $p = 0.83$. No evidence of effect modification by setting.

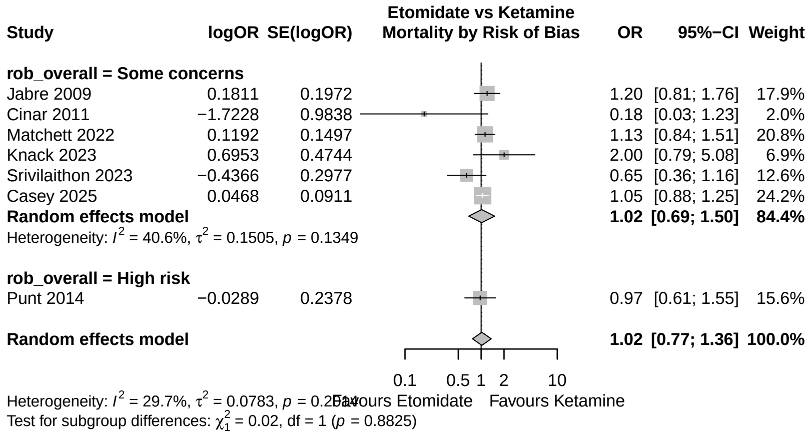

eFigure e10. Subgroup analysis for etomidate vs ketamine mortality by overall risk of bias. Test for subgroup differences: $p = 0.88$. Only one study (Punt 2014) was rated "high risk."

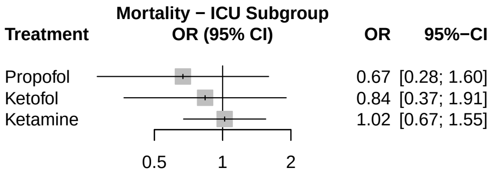

eFigure e11. Network meta-analysis restricted to ICU studies (5 studies: Cinar, Punt, Smischney, Matchett, Schmidt). This subgroup includes all four treatment nodes with a connected network.

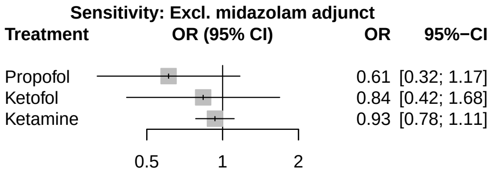

eFigure e12. Sensitivity analysis excluding studies with midazolam adjunct in the ketamine arm (Cinar 2011, Punt 2014 excluded; 7 studies remaining).

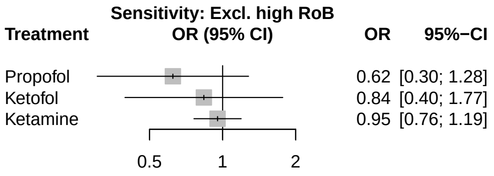

eFigure e13. Sensitivity analysis excluding high risk-of-bias studies (Punt 2014 excluded; 8 studies remaining).

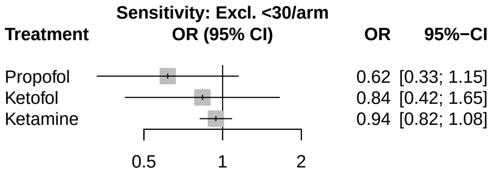

eFigure e14. Sensitivity analysis excluding small studies (<30 patients per arm; Cinar 2011 excluded; 8 studies remaining).

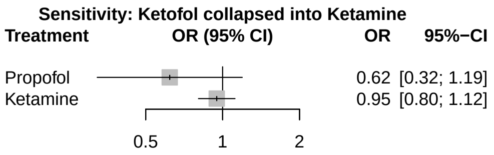

eFigure e15. Sensitivity analysis collapsing the ketofol node into ketamine (three-node network: etomidate, ketamine, propofol).

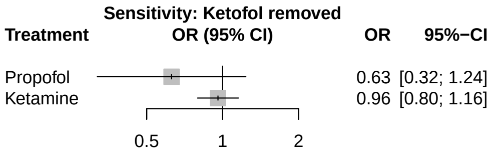

eFigure e16. Sensitivity analysis removing the ketofol node and the corresponding study (Smischney 2019 excluded; 8 studies, three-node network).

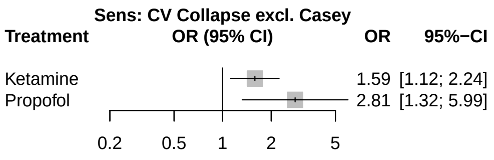

eFigure e17. Sensitivity analysis excluding Casey 2025 (RSI trial): cardiovascular collapse. Two studies remaining (Matchett 2022, Schmidt 2025). Ketamine vs etomidate OR 1.59 (1.12–2.24); finding remains significant.

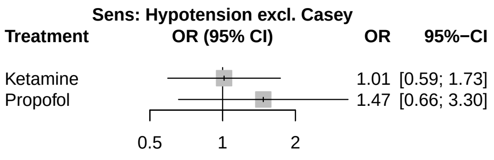

eFigure e18. Sensitivity analysis excluding Casey 2025 (RSI trial): post-induction hypotension. Three studies remaining (Knack, Srivilaithon, Schmidt). Ketamine vs etomidate OR 1.01 (0.59–1.73); finding attenuated and no longer significant.

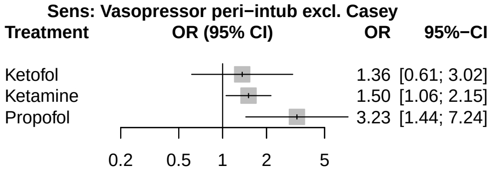

eFigure e19. Sensitivity analysis excluding Casey 2025 (RSI trial): peri-intubation vasopressor use. Four studies remaining (Cinar, Smischney, Matchett, Schmidt). Ketamine vs etomidate OR 1.50 (1.06–2.15); finding remains significant.

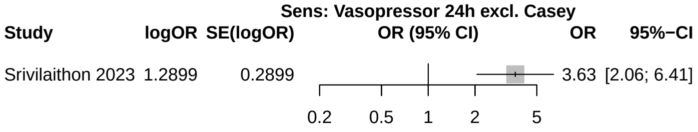

eFigure e20. Sensitivity analysis excluding Casey 2025 (RSI trial): vasopressor use at 24 hours (etomidate vs ketamine direction). One study remaining (Srivilaithon 2023). OR 3.63 (2.06–6.41), indicating substantially higher 24-hour vasopressor use with etomidate in this sepsis-only population. Heterogeneity present in the main analysis (I^2 = 93%) cannot be assessed with a single study.

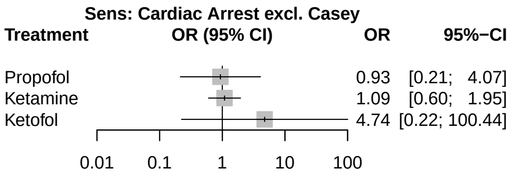

eFigure e21. Sensitivity analysis excluding Casey 2025 (RSI trial): peri-intubation cardiac arrest. Six studies remaining. Ketamine vs etomidate OR 1.09 (0.60–1.95); finding unchanged.

4 Certainty of Evidence (CINeMA Framework)

Certainty of evidence was assessed using the CINeMA framework (Confidence in Network Meta-Analysis) for key comparisons.

4.1 Primary outcome: short-term mortality

eTable e1. CINeMA assessment — Ketamine vs Etomidate, mortality (OR 0.96, 95% CI 0.80–1.16)

**Domain**	**Judgement**	**Rationale**
Within-study bias	Some concerns	All 7 contributing studies rated "some concerns" (6) or "high risk" (1, Punt). Open-label designs in 6/7 studies, but mortality is objective and the risk-of-bias subgroup analysis showed no effect modification (p = 0.88). Not downgraded.
Reporting bias	No concerns	9 studies in NMA; funnel plot shows no clear asymmetry. Most studies pre-registered.
Indirectness	No concerns	All 7 studies directly compare E and K in the target population. Settings include ED, ICU, and mixed.
Imprecision	Some concerns	95% CI (0.80–1.16) includes OR = 1.0; on an absolute scale at 30% baseline mortality, the CI corresponds to approximately −5% to +4%, which does not exclude a clinically important difference of 2–3%. **Downgraded one level.**
Heterogeneity	No concerns	$I^2 = 30\%$, $\tau^2 = 0.017$. Subgroup analyses showed no significant effect modification (setting $p = 0.83$; RoB $p = 0.88$).
Incoherence	No concerns	100% direct evidence.
**Overall certainty: MODERATE** (downgraded one level for imprecision)		

eTable e2. CINeMA assessment — Ketamine vs Propofol, mortality (OR 1.53, 95% CI 0.80–2.93)

Domain	Judgement	Rationale
Within-study bias	Some concerns	Based entirely on PROMINE (open-label, mITT excluding 15.5% including pre-consent deaths).
Reporting bias	Some concerns	Single direct study. Mortality was not the primary endpoint of PROMINE. Cannot assess small-study effects.
Indirectness	Some concerns	PROMINE used esketamine 2 mg/kg in high-severity ICU population in Brazil (hospital mortality ∼55%). May not generalize to ED, lower-acuity, or racemic ketamine.
Imprecision	Major concerns	Wide 95% CI (0.80–2.93) spanning from meaningful benefit to substantial harm. Only 175 patients.
Heterogeneity	No concerns	Single study.
Incoherence	No concerns	100% direct evidence.
**Overall certainty: LOW** (downgraded for imprecision [major] and indirectness)		

eTable e3. CINeMA assessment — Etomidate vs Propofol, mortality (OR 0.63, 95% CI 0.32–1.24; indirect)

Domain	Judgement	Rationale
Within-study bias	Some concerns	Entirely indirect: 50% from E–K studies + 50% from PROMINE. Contribution-weighted RoB: some concerns.
Reporting bias	Some concerns	Indirect comparison limits assessment.
Indirectness	Major concerns	No direct evidence. Transitivity assumption through ketamine node. E–K and K–P studies differ in setting, ketamine formulation (racemic vs esketamine), and population severity.
Imprecision	Major concerns	Wide 95% CI (0.32–1.24). Indirect estimation amplifies uncertainty.
Heterogeneity	No concerns	Network-level I^2 = 30%.
Incoherence	No concerns	Cannot assess (no direct evidence).
**Overall certainty: VERY LOW** (downgraded for bias, indirectness [major], imprecision [major])		

4.2 Key secondary outcomes (Ketamine vs Etomidate)

eTable e4. CINeMA assessment summary — Secondary outcomes, Ketamine vs Etomidate

Outcome	Bias	Report.	Indir.	Imprec.	Heter.	Incoh.	Overall
CV collapse	SC	SC	NC	NC	NC	NC	Moderate
Hypotension	SC	SC	SC	NC	NC	NC	Low
Vaso (peri)	SC	SC	SC	NC	NC	NC	Low
First-pass	SC	SC	NC	NC	NC	NC	Moderate
Cardiac arr.	SC	SC	NC	SC	NC	NC	Low

SC = some concerns; NC = no concerns. Vaso = vasopressor use; peri = peri-intubation. CV = cardiovascular. See main text for detailed rationale.

5 Data Extractions

Data were extracted independently by two reviewers (or by machine extraction with full human verification against source PDFs). Below we present the mortality data summary followed by full per-study extraction forms for all 9 included studies (N = 4,672 patients).

5.1 Summary of mortality data

Study	Year	Comparison	N	Setting	Timepoint	Arm A deaths	Arm B deaths
Jabre (KETASED)	2009	E vs K	469	Mixed	28-day	81/234	72/235
Cinar*	2011	E vs K	22	ICU	ICU	5/12	8/10
Punt*	2014	E vs SK	301	ICU	28-day	61/161	54/140
Smischney	2019	E vs KF	152	ICU	Hospital	26/73	25/79
Matchett (EvK)	2022	E vs K	791	ICU	28-day	142/396	131/395
Knack	2023	E vs K	143	ED	30-day	15/73	8/70
Srivilaithon	2023	E vs K	260	ED	28-day	25/130	35/130
Casey (RSI)	2025	E vs K	2,359	Mixed	28-day	345/1186	330/1173
Schmidt (PROMINE)	2025	P vs ESK	175	ICU	Hospital	42/84	55/91

E = etomidate; K = ketamine; SK = S-ketamine; ESK = esketamine; P = propofol; KF = ketofol. Arm A = first-named treatment; Arm B = second-named treatment.

*Ketamine arm included midazolam adjunct — flagged for sensitivity analysis.

5.2 Per-study extraction forms

5.2.1 Study 1: Jabre 2009 (KETASED)

Identification and design. Jabre P et al., *The Lancet* 2009. France. KETASED trial (NCT00440102). Funded by French Ministry of Health (PHRC 2006 AOM06103). Prospective, randomized, single-blind, parallel-group trial across 12 EMS/ED centers (65 receiving ICUs). April 2007 to February 2008. Single-blind: enrolling emergency physicians aware of allocation; ICU nurses and intensivists masked.

	Etomidate	Ketamine
Randomized, n	328	327
Analyzed (mITT), n	234	235
Age, mean (SD)	57 (18)	59 (19)
Male, n (%)	147 (63%)	133 (57%)
SAPS II, mean (SD)	51.2 (18.3)	50.5 (17.4)
GCS, median (range)	6 (3–15)	7 (3–15)
Baseline SBP, mean (SD) mmHg	132 (38)	128 (32)
Sepsis, n (%)	41 (18%)	35 (15%)

Population. **Inclusion:** Adults ≥18 years requiring emergency orotracheal intubation with sedation.
Exclusion: Cardiac arrest; contraindications to succinylcholine, ketamine, or etomidate; known pregnancy.
mITT exclusions: 186/655 (28%) excluded (ICU discharge <3 days, pre-hospital death, consent withdrawal, missing data).

Interventions. Etomidate 0.3 mg/kg vs ketamine 2 mg/kg, both IV bolus. Succinylcholine 1 mg/kg in both arms. No co-induction agents. Post-intubation: standardized midazolam 0.1 mg/kg/h + fentanyl. Strict RSI protocol (fixed doses).

Outcome	Etomidate	Ketamine	Effect
28-day mortality	81/234 (35%)	72/235 (31%)	OR 1.2 (0.8–1.8), $p = 0.36$
Cardiac arrest (peri-intub.)	7/234 (3%)	4/235 (2%)	RD 1.3 pp
Change in SBP, median (IQR)	5 (−11 to 30)	10 (−10 to 33)	−5 (−13 to 2)
Catecholamine support (ICU)	137/234 (59%)	120/235 (51%)	RD 7.5 pp, $p = 0.10$
Difficult intubation (IDS >5)	24 (10%)	20 (9%)	—

Outcomes.

Domain	Judgement	Support
D1: Randomization	Low risk	Computerized RNG, blocks of 4, stratified by center, sealed drug boxes.
D2: Deviations	Some concerns	Single-blind: emergency physicians aware; ICU staff masked.
D3: Missing data	Some concerns	mITT excluded 28%. ITT (n = 650) consistent ($p = 0.54$).
D4: Measurement	Low risk	28-day mortality is objective.
D5: Reporting	Low risk	Pre-registered (NCT00440102).
**Overall**	**Some concerns**	Partial blinding + substantial mITT exclusions.

Risk of bias (RoB 2).

Notes. Primary endpoint was maximum SOFA score. Adrenal insufficiency significantly higher with etomidate: OR 6.7 (95% CI 3.5–12.7). Prehospital enrollment (EMS setting). For NMA: 81/234 (etomidate) vs 72/235 (ketamine).

5.2.2 Study 2: Cinar 2011

Identification and design. Cinar O et al., *J Turkish Soc Intensive Care* 2011. Turkey. No trial registration. Single-center (Baskent University Hospital, Ankara). Double-blind RCT: identical syringes prepared by uninvolved clinician; all intubations by single operator. Ethics approval KA09/27.

	Etomidate	Ketamine + Midazolam
Analyzed, n	12	10
Age, mean ± SD	63.8 ± 20.3	72.5 ± 7.8
Female, n (%)	7 (58%)	4 (40%)
APACHE II, mean ± SD	28.7 ± 5.4	32.1 ± 7.0

Population. Inclusion: ICU patients requiring emergency intubation (GCS <8, respiratory failure).
Exclusion: Cardiac arrest; prior corticosteroids; adrenal insufficiency; psychotic disorder.

Interventions. Etomidate 0.3 mg/kg vs ketamine 2 mg/kg + midazolam 0.03 mg/kg (combined in same syringe), both IV. No NMBA used in either arm. All intubations by same operator. Strict protocol.

Outcome	Etomidate	Ket + Midaz	Effect
ICU mortality	5/12 (42%)	8/10 (80%)	$p = 0.099$
Vasopressor (peri-intub.)	2/12 (17%)	2/10 (20%)	$p = 1.0$
Cardiac arrest (peri-intub.)	1/12	1/10	—

Outcomes.

Domain	Judgement	Support
D1: Randomization	Some concerns	Web-based RNG. Very small sample; APACHE II imbalance.
D2: Deviations	Low risk	Double-blind (identical syringes, single operator).
D3: Missing data	Low risk	All 22 accounted for.
D4: Measurement	Low risk	Mortality is objective.
D5: Reporting	Some concerns	No trial registration. Mortality was secondary.
**Overall**	**Some concerns**	Small unregistered trial. Double-blind is a strength.

Risk of bias (RoB 2).

Notes. **Flagged for sensitivity analysis:** ketamine arm received midazolam adjunct. Extremely small trial (n = 22). No NMBA used. ICU mortality (only available timepoint). For NMA: 5/12 (etomidate) vs 8/10 (ketamine).

5.2.3 Study 3: Punt 2014

Identification and design. Punt CD, Dormans TPJ et al., *Neth J Crit Care* 2014. The Netherlands. ISRCTN39347168. Single-center cluster-randomized trial (Atrium Medical Centre, Heerlen; 3 ICU units, 21 beds). Open-label. April 2008 to end of 2009. Cluster design: 2 units used etomidate and 1 used S-ketamine for 10 months, then reversed.

	Etomidate	S-ketamine + Midazolam
Analyzed, n	161	140
Age, mean (SD)	66 (14)	67 (13)
Male, n (%)	95 (59%)	89 (64%)
APACHE II, mean (SD)	25 (7)	24 (7)
Sepsis, n	58	54

Population. **Inclusion:** All critically ill adults intubated in participating ICU units.
Exclusion: Already intubated before ICU admission; received etomidate <72h before enrollment.

Interventions. Etomidate 0.2–0.3 mg/kg vs S-ketamine 0.5 mg/kg + midazolam 2.5 mg, both IV. Rocuronium in both arms. Pragmatic protocol.

Outcome	Etomidate	S-ket + Midaz	Effect
28-day mortality	61/161 (38%)	54/140 (39%)	$p = 0.998$
NE hours (72h), mean (SD)	26 (31)	29 (29)	$p = 0.389$
ICU LOS, days, mean (SD)	16 (25)	19 (27)	$p = 0.318$

Outcomes.

Domain	Judgement	Support
D1: Randomization	High risk	Cluster-randomized by ICU unit; allocation predictable.
D2: Deviations	Some concerns	Open-label. Midazolam only in S-ketamine arm.
D3: Missing data	Low risk	301 analyzed with clear accounting.
D4: Measurement	Low risk	28-day mortality is objective.
D5: Reporting	Low risk	Registered (ISRCTN39347168). Mortality was primary.
**Overall**	**High risk**	Cluster design without individual concealment; systematic co-intervention difference.

Risk of bias (RoB 2).

Notes. **Flagged for sensitivity analysis:** S-ketamine arm received midazolam 2.5 mg. S-ketamine mapped to Ketamine node. Cluster-randomized design is a major limitation. For NMA: 61/161 (etomidate) vs 54/140 (S-ketamine).

5.2.4 Study 4: Smischney 2019 (KEEP PACE)

Identification and design. Smischney NJ et al., *J Trauma Acute Care Surg* 2019. United States. KEEP PACE trial (NCT02105415). Funded by Dept of Anesthesiology, Mayo Clinic; CTSA grant. Single-center (Mayo Clinic, Rochester, MN). Open-label intervention; outcome adjudicators and statistician blinded. July 2014 to October 2017.

	Etomidate	Ketofol
Randomized, n	76	84
Analyzed, n	73	79
Age, mean ± SD	60.0 ± 18.3	62.1 ± 17.2
Male, n (%)	38 (52%)	48 (61%)
APACHE III, mean ± SD	86.7 ± 30.4	85.8 ± 31.3
Baseline MAP, mean ± SD mmHg	82.8 ± 17.9	80.9 ± 13.3
On vasopressors, n (%)	21 (29%)	17 (22%)
Sepsis, n (%)	57 (78%)	61 (77%)

Population. **Inclusion:** Adults ≥18 years admitted to ICU requiring emergent intubation. **Exclusion:** Cardiac arrest; ICP pathology; chronic opioid dependence; bipolar/schizophrenia; egg allergy; weight >140 or <30 kg.

Interventions. Etomidate 0.15 mg/kg (reduced dose) vs ketofol (ketamine 0.5 mg/kg + propofol 0.5 mg/kg, single syringe). Fentanyl 50 μg IV standard in both arms. Rescue doses allowed. Succinylcholine or rocuronium per clinician choice. Pragmatic protocol.

Outcome	Etomidate	Ketofol	Effect
Hospital mortality	26/73 (36%)	25/79 (32%)	$p = 0.605$
Vasopressor 5 min	13/73 (18%)	18/79 (24%)	$p = 0.446$
Vasopressor 15 min–24h	57/73 (78%)	64/79 (81%)	$p = 0.691$
Adrenal insuff. 3–5h	13/16 (81%)	5/13 (38%)	$p = 0.027$

Outcomes.

Domain	Judgement	Support
D1: Randomization	Low risk	Computer-generated, stratified by unit and shock, sealed envelopes.
D2: Deviations	Some concerns	Open-label. Co-interventions noncontrolled.
D3: Missing data	Low risk	8/160 (5%) withdrew consent. 152 analyzed.
D4: Measurement	Low risk	Hospital mortality is objective.
D5: Reporting	Low risk	Pre-registered (NCT02105415). DSMB oversight.
**Overall**	**Some concerns**	Open-label with noncontrolled co-interventions.

Risk of bias (RoB 2).

Notes. Only trial providing the **ketofol–etomidate** edge in the NMA. Both drug doses reduced from standard. Primary endpoint was MAP at 5 min. For NMA: 26/73 (etomidate) vs 25/79 (ketofol).

5.2.5 Study 5: Matchett 2022 (EvK)

Identification and design. Matchett G et al., *Intensive Care Med* 2022. United States. EvK trial (NCT02643381). EFIC trial at UT-Southwestern Medical Center, Dallas. Single-center, open-label RCT. June 2016 to September 2020. No external funding.

	Etomidate	Ketamine
Randomized, n	400	401
Analyzed, n	396	395
Age, mean (SD)	55.8 (15.2)	55.4 (16)
Female, n (%)	153 (38.6%)	150 (38%)
Indication: shock, n (%)	189 (47.7%)	174 (44.1%)
Baseline SBP, mean (SD) mmHg	120.7 (32.2)	120.3 (29.5)
Sepsis, n (%)	136 (34.3%)	136 (34.4%)

Population. **Inclusion:** Adults ≥18 years requiring emergency endotracheal intubation. **Exclusion:** Cardiac arrest; pregnant; previously enrolled; known allergy.

Interventions. Etomidate 0.2–0.3 mg/kg (median actual 0.2 mg/kg) vs ketamine 1–2 mg/kg (median actual 1.1 mg/kg). Rocuronium (80%) or succinylcholine (18%). Semi-standardized airway bundle protocol.

Outcome	Etomidate	Ketamine	Effect
Day 28 mortality	142/396 (35.9%)	131/395 (33.2%)	RD −2.7 pp (−9.3 to 3.9)
Day 7 mortality	90/396 (22.7%)	59/395 (14.9%)	RD −7.8 pp (−13 to −2.4)†
CV collapse	69/396 (17.4%)	99/395 (25.1%)	RD −7.6 pp (−13 to −2)
First-pass success	357/391 (91.3%)	355/389 (91.3%)	RD 0 pp
Vasopressor Days 1–4	235/396 (59.3%)	213/395 (53.9%)	RD 5.4 pp (−1.5 to 12.3)
Cardiac arrest (post-induction CPR)‡	13/345 (3.8%)	18/354 (5.1%)	—
Vasopressor bolus (peri-intubation)‡	65/345 (18.8%)	92/354 (26.0%)	—

†Day 7 was the original primary endpoint; significantly favored ketamine.

‡Denominators reduced (345/354) due to ∼12% missing data from nurses' code sheets (unavailable for 51 etomidate, 41 ketamine patients).

Outcomes.

Domain	Judgement	Support
D1: Randomization	Low risk	Computer-generated, blocks of 8, sealed envelopes.
D2: Deviations	Some concerns	Completely open-label.
D3: Missing data	Low risk	10/801 (1.2%) withdrawals.
D4: Measurement	Low risk	Day 28 mortality is objective.
D5: Reporting	Low risk	Pre-registered. SAP finalized before analysis.
**Overall**	**Some concerns**	Due to open-label design.

Risk of bias (RoB 2).

Notes. CV collapse higher with ketamine (25.1% vs 17.4%), consistent with Casey 2025. Low actual ketamine dose (median 1.1 mg/kg). Trial closed early by DSMB. For NMA: 142/396 (etomidate) vs 131/395 (ketamine).

5.2.6 Study 6: Knack 2023

Identification and design. Knack SKS et al., *J Emerg Med* 2023. United States. NCT01823328. EFIC trial at Hennepin County Medical Center, Minneapolis. Single-center, partially blinded (ED team aware; ICU team blinded). September 2013 to November 2015. Full publication of Driver 2014 conference abstract.

	Etomidate	Ketamine
Analyzed (ITT), n	73	70
Age, median (IQR)	49 (31–58)	50 (32–65)
Male, n (%)	49 (67%)	42 (60%)
GCS, median (IQR)	8 (6–11)	7 (6–12)
Baseline SBP, median (IQR)	140 (119–167)	139 (128–161)
Sepsis, n (%)	19 (26%)	10 (14%)

Population. **Inclusion:** Adults ≥18 years undergoing RSI in the ED, critically ill.
Exclusion: Increased HR/BP hazardous; suspected elevated ICP; known allergy.

Interventions. Etomidate 0.3 mg/kg vs ketamine 2 mg/kg. Succinylcholine in ∼90%. Pragmatic protocol (devices at physician discretion).

Outcome	Etomidate	Ketamine	Effect
30-day mortality	15/73 (21%)	8/70 (11%)	RD −9 pp (−21 to 3)
Hypotension (SBP <90) in ED	19/72 (26%)	19/67 (28%)	RD 2 pp (−13 to 17)
First-pass success	65/73 (89%)	66/70 (94%)	RD 5 pp (−4 to 13)

Outcomes.

Domain	Judgement	Support
D1: Randomization	Low risk	Computer-generated, permuted blocks, sealed opaque envelopes.
D2: Deviations	Some concerns	ED unblinded; ICU blinded. 5 crossovers.
D3: Missing data	Low risk	All 143 in ITT analysis.
D4: Measurement	Low risk	30-day mortality is objective.
D5: Reporting	Some concerns	Primary outcome changed mid-trial (from mortality to max SOFA).
**Overall**	**Some concerns**	Partially blinded; endpoint changed mid-trial.

Risk of bias (RoB 2).

Notes. Small trial (N = 143). Young cohort (median age 50). 10% absolute mortality difference (not significant). For NMA: 15/73 (etomidate) vs 8/70 (ketamine).

5.2.7 Study 7: Srivilaithon 2023

Identification and design. Srivilaithon W et al., *Scientific Reports* 2023. Thailand. TCTR20210213001 (Thai registry, **retrospectively registered**). Single-center (Thammasat University Hospital). Single-blind: enrolling physician aware but was not the intubating physician; outcome assessors blinded. March 2019 to December 2020.

	Etomidate	Ketamine
Analyzed, n	130	130
Age, mean (SD)	73.2 (12.6)	70.5 (14.9)
Male, n (%)	77 (59.2%)	76 (58.5%)
qSOFA, mean (SD)	2.2 (0.4)	2.1 (0.3)
Baseline SBP, mean (SD) mmHg	112.9 (30.7)	118.1 (32.5)
Lactate, median (IQR) mmol/L	3.6 (2.4–7.6)	3.2 (2.2–5.4)
Sepsis, n (%)	130 (100%)	130 (100%)

Population. **Inclusion:** Adults ≥18 years with suspected sepsis (Sepsis-3) requiring emergency intubation in the ED.
Exclusion: Cardiac arrest; DNR; adrenal insufficiency; severe hypertension; elevated ICP.

Interventions. Etomidate 0.2–0.3 mg/kg vs ketamine 1–2 mg/kg. Succinylcholine 1.5 mg/kg (NMBA use: 65% E vs 77% K, $p = 0.04$). Semi-standardized protocol.

Outcome	Etomidate	Ketamine	Effect
28-day mortality	25/130 (19.2%)	35/130 (26.9%)	RD 7.7 pp (−2.5 to 17.9)
Cardiac arrest (peri-intub.)	2/130 (1.5%)	2/130 (1.5%)	$p = 1.0$
Hypotension (SBP <90 / MAP <65)	15/130 (11.5%)	14/130 (10.8%)	RD 0.7 pp
Vasopressor use 24h	57/130 (43.9%)	23/130 (17.7%)	RD 26.2 pp, $p < 0.001$
First-pass success	116/130 (89.2%)	114/130 (87.7%)	$p = 0.846$

Outcomes.

Domain	Judgement	Support
D1: Randomization	Low risk	Computer-generated, blocks of 4, sealed envelopes.
D2: Deviations	Some concerns	Single-blind. NMBA use differed ($p = 0.04$).
D3: Missing data	Low risk	All 260 analyzed, 0 lost.
D4: Measurement	Low risk	28-day mortality is objective.
D5: Reporting	Some concerns	Retrospectively registered (Feb 2021; trial 2019–2020).
**Overall**	**Some concerns**	Single-blind with differential NMBA use; retrospective registration.

Risk of bias (RoB 2).

Notes. Sepsis-only population (100%). Elderly (mean age ∼72). Vasopressor 24h much higher with etomidate (44% vs 18%, $p < 0.001$) — likely reflects adrenal suppression. For NMA: 25/130 (etomidate) vs 35/130 (ketamine).

5.2.8 Study 8: Casey 2025 (RSI Trial)

Identification and design. Casey JD et al., *New Engl J Med* 2025. United States. RSI trial (NCT05277896). Funded by PCORI and NHLBI. Pragmatic, multicenter, unblinded RCT. 14 sites (6 EDs + 8 ICUs) in 6 medical centers. EFIC trial. April 2022 to August 2025.

	Etomidate	Ketamine
Randomized, n	1,189	1,176
Analyzed, n	1,186	1,173
Age, median (IQR)	60 (44–69)	60 (45–69)
Female, n (%)	492 (41.4%)	498 (42.3%)
APACHE II, median (IQR)	18 (13–24)	18 (13–24)
SBP at induction, median (IQR)	127 (110–148)	127 (110–147)
On vasopressors, n (%)	274 (23.0%)	246 (20.9%)
Sepsis/septic shock, n (%)	565 (47.5%)	539 (45.8%)
Location — ED, n (%)	655 (55.1%)	663 (56.4%)

Population. Inclusion: Adults ≥18 years, critically ill, undergoing tracheal intubation with anesthesia induction.
Exclusion: Pregnant; prisoners; primary trauma; clinician determined specific agent necessary.

Interventions. Etomidate 0.2–0.3 mg/kg (median 0.28) vs ketamine 1.0–2.0 mg/kg (median 1.6), dose at clinician choice. Rocuronium (∼69%) or succinylcholine (∼31%). Pragmatic protocol.

Outcome	Etomidate	Ketamine	Effect
In-hospital death by 28d	345/1186 (29.1%)	330/1173 (28.1%)	adj. RD −0.8 pp (−4.5 to 2.9)
CV collapse	202/1189 (17.0%)	260/1176 (22.1%)	RD 5.1 pp (1.9 to 8.3)
SBP <65	64 (5.5%)	73 (6.4%)	—
New/incr. vasopressor	189 (15.9%)	251 (21.3%)	RD 5.4 pp (2.3 to 8.6)
Cardiac arrest	10 (0.8%)	12 (1.0%)	—
Hypotension (SBP <80)	123/∼1159 (10.6%)	164/∼1138 (14.4%)	RD 3.8 pp (1.1 to 6.5)
Vaso peri-intub.	189/1189 (15.9%)	251/1176 (21.3%)	RD 5.4 pp (2.3 to 8.6)
Vaso at 24h	458/∼1082 (42.3%)	420/∼1079 (38.9%)	RD −3.4 (−7.5 to 0.7)
First-pass success	1029/∼1187 (86.7%)	1005/∼1173 (85.7%)	RD −1.0 pp

Outcomes.

Domain	Judgement	Support
D1: Randomization	Low risk	Permuted blocks, stratified by site, concealed envelopes.
D2: Deviations	Some concerns	Unblinded. Adherence >99%. Could influence co-interventions.
D3: Missing data	Low risk	Only 6/2,365 (0.3%) withdrew.
D4: Measurement	Low risk	Mortality is objective.
D5: Reporting	Low risk	Pre-registered. Protocol and SAP published before analysis.
**Overall**	**Some concerns**	Open-label. Mortality unlikely influenced by lack of blinding.

Risk of bias (RoB 2).

Notes. Largest trial (N = 2,365; ∼48% of total sample). CV collapse higher with ketamine (22.1% vs 17.0%), driven by vasopressor escalation. 24h vasopressor trend reversed (favoring ketamine). Ventricular tachycardia (post hoc): K 1.0% vs E 0.2%. For NMA: 345/1186 (etomidate) vs 330/1173 (ketamine).

5.2.9 Study 9: Schmidt 2025 (PROMINE)

Identification and design. Schmidt RC et al., *Under review* 2025. Brazil. PROMINE trial (NCT05092152). Funded by CNPq; esketamine and propofol provided by Cristália. Investigator-initiated, randomized, open-label, parallel-group. 2 hospitals (8 ICUs, 93 beds). Outcome assessors, data collectors, and statisticians blinded. October 2021 to October 2023.

	Propofol	Esketamine
Randomized, n	102	105
Analyzed (mITT), n	84	91
Age, mean (SD)	61 (16)	62 (17)
Male, n (%)	46 (55%)	47 (52%)
SAPS 3, mean (SD)	60 (15)	66 (17)
SOFA, mean (SD)	7 (4)	8 (4)
Baseline MAP, mean (SD) mmHg	91 (16.6)	90 (16.2)
On vasopressors, n (%)	31 (37%)	30 (33%)
Heart failure, n (%)	11 (13%)	27 (30%)
Infection, n (%)	54 (64%)	61 (67%)

Population. **Inclusion:** Adults ≥18 with indication for orotracheal intubation in participating ICUs.
Exclusion: Intubation during cardiac arrest; ICP pathology; bradycardia <50 bpm; pregnancy; known allergy.

mITT exclusions: 32/207 (15.5%) — never intubated, consent unavailable before death, refused consent.

Interventions. Propofol 1.5 mg/kg vs esketamine 2 mg/kg ($\approx$ 4 mg/kg racemic equivalent), both IV bolus (∼10 sec). Premedication: fentanyl in 95%. Rocuronium 1.2 mg/kg in ∼98%. Strict RSI protocol with standardized checklist.

Outcome	Propofol	Esketamine	Effect
Hospital mortality	42/84 (50.0%)	55/91 (60.4%)	OR 1.21 (0.92–1.58)
7-day mortality	20/84 (23.8%)	30/91 (33.0%)	OR 1.38 (0.86–2.24)
CV collapse	28/84 (33%)	20/91 (22%)	OR 0.56 (0.28–1.10)
SBP ≤65 (2 min)	5/84 (6%)	6/91 (7%)	—
Cardiac arrest (1h)	4/84 (4.8%)	5/91 (5.5%)	—
Vasopressor increase (2 min)	25/84 (29.7%)	15/91 (16.5%)	—
Severe hypotension (SBP <80, 1h)	39/84 (46.4%)	34/91 (37.4%)	OR 0.80 (0.57–1.14)
Lowest MAP 10 min, median (IQR)	60 (48–72)	66 (55–79)	adj. MD 6.0 (−0.0 to 11.9)
First-pass success	62/84 (74%)	69/91 (76%)	—

Outcomes.

Domain	Judgement	Support
D1: Randomization	Low risk	Centralized REDCap, variable blocks, stratified by site and vasopressor use.
D2: Deviations	Some concerns	Open-label. ICU team knew allocation.
D3: Missing data	Some concerns	mITT excluded 15.5%, including 15 pre-consent deaths.
D4: Measurement	Low risk	Hospital mortality is objective.
D5: Reporting	Some concerns	Primary was MAP. Hospital mortality was exploratory.
**Overall**	**Some concerns**	Open-label; mITT excluding 15% including pre-consent deaths.

Risk of bias (RoB 2).

Notes. **Only trial comparing ketamine (esketamine) vs propofol** — provides the critical propofol edge for the NMA. Esketamine at 2 mg/kg mapped to Ketamine node. Very high mortality (∼55% overall). No 28-day mortality reported; hospital mortality (censored at day 60) used. Heart failure imbalance (30% esketamine vs 13% propofol) could confound mortality. First-pass success

lower ($\sim$75%) reflecting resident-heavy ICU intubation. For NMA: 42/84 (propofol) vs 55/91 (esketamine/ketamine).

5.3 Risk of bias summary across studies

Study	D1: Rand.	D2: Dev.	D3: Miss.	D4: Meas.	D5: Report.	Overall
Jabre 2009	Low	SC	SC	Low	Low	SC
Cinar 2011	SC	Low	Low	Low	SC	SC
Punt 2014	High	SC	Low	Low	Low	**High**
Smischney 2019	Low	SC	Low	Low	Low	SC
Matchett 2022	Low	SC	Low	Low	Low	SC
Knack 2023	Low	SC	Low	Low	SC	SC
Srivilaithon 2023	Low	SC	Low	Low	SC	SC
Casey 2025	Low	SC	Low	Low	Low	SC
Schmidt 2025	Low	SC	SC	Low	SC	SC

SC = some concerns. D1 = bias arising from randomization process; D2 = bias due to deviations from intended interventions; D3 = bias due to missing outcome data; D4 = bias in measurement of the outcome; D5 = bias in selection of reported result. One study rated high risk of bias overall (Punt 2014); eight studies rated “some concerns” (primarily open-label or partially blinded designs). No studies were rated low risk of bias overall.

6 Expression of Concern: Possible Data Duplication (Agarwal 2025)

6.1 Papers under comparison

- **Srivilaithon W et al.** Clinical outcomes after a single induction dose of etomidate versus ketamine for emergency department sepsis intubation: a randomized controlled trial. *Sci Rep* 2023; 13:6362. (N = 260, 130/arm; Thailand)
- **Agarwal D, Goyal C.** Comparative Outcomes of Etomidate versus Ketamine for Emergency Intubation in Septic Patients: A Randomized Controlled Trial. *SSR Inst Int J Life Sci* 2025; 11(5):8348–8355. (N = 80, 40/arm; India)

6.2 Context

During screening for this systematic review, extensive overlap was identified between the summary statistics reported in these two papers. Despite being conducted at different institutions in different countries with different sample sizes, the reported data are remarkably similar across baseline characteristics, intubation conditions, and outcomes. The findings below are presented for consideration.

6.3 Baseline characteristics

Variable	Srivilaithon 2023 (N=260)	Agarwal 2025 (N=80)	Observation
Male gender, n (%)	153 (58.9%)	47 (58.8%)	Near-identical
Age, mean ± SD	71.9 ± 13.9	71.9 ± 13.9	Identical
Diabetes mellitus, n (%)	107 (41.2%)	33 (41.3%)	Near-identical
Hypertension, n (%)	156 (60.0%)	48 (60.0%)	Identical
Stroke, n (%)	69 (26.5%)	21 (26.3%)	Near-identical
CKD, n (%)	31 (11.9%)	10 (12.5%)	Near-identical
COPD/asthma, n (%)	20 (7.7%)	6 (7.5%)	Near-identical
Resp. tract infection, n (%)	187 (71.9%)	58 (72.5%)	Near-identical
SBP, mean ± SD (mmHg)	115.5 ± 31.7	115.5 ± 31.7	Identical
Pulse rate, mean ± SD (bpm)	107.2 ± 24.9	107.2 ± 24.9	Identical
O_2 sat, median (IQR) (%)	92 (83, 98)	92 (83–98)	Identical
qSOFA, mean ± SD	2.2 ± 0.4	2.2 ± 0.4	Identical
Delta SOFA, mean ± SD	4.8 ± 1.9	4.8 ± 1.9	Identical
Lactate, median (IQR) (mmol/L)	3.3 (2.4, 6.5)	3.3 (2.4–6.5)	Identical
IV antibiotic pre-rand., n (%)	214 (82.3%)	66 (82.5%)	Near-identical
IV fluid, median (IQR) (mL)	1000 (600, 1500)	1000 (600–1500)	Identical

6.4 Within-arm baseline values

Variable	Sriv. Etom (N=130)	Agar. Etom (N=40)	Sriv. Ket (N=130)	Agar. Ket (N=40)
Age, mean ± SD	73.2 ± 12.6	73.2 ± 12.6	70.5 ± 14.9	70.5 ± 14.9
SBP, mean ± SD	112.9 ± 30.7	112.9 ± 30.7	118.1 ± 32.5	118.1 ± 32.5
Pulse, mean ± SD	108.8 ± 24.5	108.8 ± 24.5	105.6 ± 25.2	105.6 ± 25.2
O_2 sat, med (IQR)	92 (84, 98)	92 (84–98)	92 (83, 98)	92 (83–98)
qSOFA, mean ± SD	2.2 ± 0.4	2.2 ± 0.4	2.1 ± 0.3	2.1 ± 0.3
ΔSOFA, mean ± SD	4.6 ± 1.9	4.6 ± 1.9	4.9 ± 1.9	4.9 ± 1.9
Lactate, med (IQR)	3.6 (2.4, 7.6)	3.6 (2.4–7.6)	3.2 (2.2, 5.4)	3.2 (2.2–5.4)

All within-arm continuous summary statistics are identical to the decimal across the two papers.

6.5 Intubation conditions

Variable	Srivilaithon Etom / Ket	Agarwal Etom / Ket	Observation
NMB use	64.6% / 76.9%	65.0% / 77.5%	Near-identical
Post-intub. SBP, mean ± SD	132.9 ± 46.9 / 142.6 ± 37.9	132.9 ± 46.9 / 142.6 ± 37.9	Identical
Post-intub. pulse, mean ± SD	116.6 ± 23.5 / 112.5 ± 21.5	116.6 ± 23.5 / 112.5 ± 21.5	Identical
Post-intub. O_2 sat, med (IQR)	100 (100, 100) / 100 (100, 100)	100 (100–100) / 100 (100–100)	Identical

6.6 Outcomes

**Outcome**	**Srivilaithon Etom / Ket (*p*)**	**Agarwal Etom / Ket (*p*)**	**Observation**
24-h survival	91.5% / 96.2% (0.097)	92.5% / 95.0% (0.09)	Near-identical
7-day survival	87.7% / 87.7% (0.574)	87.5% / 87.5% (0.57)	Near-identical
28-day survival	80.8% / 73.1% (0.092)	80.0% / 72.5% (0.09)	Near-identical
Cardiac arrest	1.5% / 1.5% (1.0)	2.5% / 2.5% (1.0)	Near-identical
Hypotension	11.5% / 10.8% (0.843)	12.5% / 10.0% (0.84)	Near-identical
Vaso. 24h	43.9% / 17.7% (<0.001)	45.0% / 17.5% (<0.001)	Near-identical
Corticosteroid	14.6% / 5.4% (0.012)	15.0% / 5.0% (0.01)	Near-identical
Fluid 3h, med (IQR)	1000 (600, 1500)	1000 (600–1500)	Identical

6.7 Note on p-values

With a sample size of 80 rather than 260, confidence intervals for the same effect sizes would be expected to be approximately 80% wider (ratio of $\sqrt{260}/\sqrt{80} \approx 1.8$). P-values should therefore be substantially larger. The near-identical p-values reported in the two papers are difficult to reconcile with independently collected data of different sample sizes.

6.8 Summary

The degree of overlap between the two papers across all reported variables—including overall and within-arm continuous statistics, categorical proportions, and p-values—raises concerns about possible data duplication. The corresponding author of the original study (Srivilaithon) has been notified. The Agarwal 2025 paper has been excluded from this systematic review.

Prepared by Fernando Zampieri, March 2026.

7 Study Protocol

The full study protocol (PROSPERO CRD420251251225) is reproduced on the following pages.

Etomidate, ketamine, and propofol for rapid sequence intubation in critically ill adults:
Protocol for a systematic review and network meta-analysis of randomized trials

Fernando G. Zampieri Raysa C Schmidt Bruno AMP Besen
Fernando JDS Ramos Flávio GR Freitas Flávia R. Machado
for the PROMINE Investigators

December 10, 2025

Registration

This protocol will be registered in the International Prospective Register of Systematic Reviews (PROSPERO). The registration ID will be added when available.

1 Background

Emergency tracheal intubation in critically ill adults is a high-risk procedure in which the choice of induction agent may substantially affect hemodynamic stability and patient outcomes. Etomidate, ketamine, and propofol are widely used hypnotic agents for rapid sequence induction (RSI), and ketamine–propofol combinations ("ketofol") are also used in some settings. These agents differ in their cardiovascular effects, and clinicians often select them based on perceived hemodynamic profiles rather than comparative effectiveness data.

Recent large randomized trials provide new, high-quality evidence that has not yet been synthesized within a unified framework. Existing systematic reviews have focused mainly on pairwise comparisons and have not integrated all relevant agents using network meta-analysis (NMA). Relevant new trials incluse Schmidt et al. [2025], Casey et al. [2025].

A comprehensive synthesis of randomized evidence is needed to clarify the comparative safety and effectiveness of induction agents used for emergency tracheal intubation in critically ill adults, particularly with respect to hemodynamic instability and mortality.

2 Objectives

The objectives of this review are:

1. To compare the effects of etomidate, ketamine, propofol, and ketofol on outcomes of emergency tracheal intubation in critically ill adults.

2. To determine the relative impact of these agents on short-term mortality, post-induction hypotension, cardiovascular collapse, vasopressor initiation, and first-pass intubation success.

3. To use network meta-analysis to estimate comparative effectiveness across all eligible induction agents using both direct and indirect randomized evidence.

4. To incorporate new randomized data (PROMINE and RSI) to provide an up-to-date assessment of induction agent performance.

5. To explore sources of heterogeneity, including clinical setting (emergency department, intensive care unit, prehospital), baseline hemodynamics, and co-interventions, if possible.

3 Methods

3.1 Eligibility criteria

3.1.1 Population

The following inclusion and exclusion criteria will be applied.

Inclusion

- Adults (typically $\geq$16 or $\geq$18 years, as defined by individual studies).
- Critically ill or acutely ill adults undergoing *emergency* or *rapid sequence* tracheal intubation.
- Intubation performed in emergency departments, intensive care units, acute hospital wards, or prehospital / EMS settings.

Exclusion

- Elective or planned operating-room intubations.
- Procedural sedation without tracheal intubation.
- Obstetric anesthesia (for example, cesarean section induction).
- Pediatric studies in which adult data cannot be isolated.
- Studies focused on sedation after intubation rather than induction.
- Elective surgical populations without acute critical illness.

3.1.2 Interventions and comparators

Eligible induction agents

- Etomidate.
- Ketamine.
- Propofol.

- Ketofol (ketamine–propofol combination).

Trials must randomize the hypnotic agent administered immediately prior to laryngoscopy for emergency or rapid sequence tracheal intubation. Any eligible agent may serve as comparator. Co-interventions (for example, opioids, neuromuscular blockers, preoxygenation strategies) are allowed if applied similarly across arms.

Excluded interventions

- Trials in which the randomized intervention is not the hypnotic induction agent (for example, neuromuscular blocker, airway device, fluid strategy).
- Sedative agents used solely for post-intubation ICU sedation.
- Agents not used in emergency RSI in critically ill adults (for example, remimazolam or ciprofol in elective anesthesia).

3.1.3 Outcomes

Trials must report at least one of the following outcomes or provide sufficient data to derive it.

Primary outcome

- **Short-term mortality**: in-hospital or 28–30 day mortality (the measure closest to 28–30 days will be used). The effect measure will be the odds ratio (OR) with 95% confidence interval (CI).

Secondary outcomes

- **Cardiovascular collapse**: composite including hypotension, new or increased vasopressor requirement, or peri-intubation cardiac arrest, as defined by individual studies. Where reported, individual components will be extracted and analyzed separately. Effect measure: OR with 95% CI.
- **Post-induction hypotension**: blood pressure below study-defined thresholds or a major decline from baseline within 0–15 minutes after induction. Effect measure: OR with 95% CI.
- **Vasopressor initiation or escalation**: new or increased vasopressor requirement. Data will be extracted at multiple time points where available (up to 30 minutes, 1 hour, and 24 hours after induction). Effect measure: OR with 95% CI.
- **First-pass intubation success**: successful endotracheal tube placement on the first laryngoscopy attempt. Effect measure: OR with 95% CI.

Outcomes reported at different time points will be analyzed separately and not pooled across time windows.

No other outcomes will be analyzed.

3.1.4 Study designs

Inclusion

- Randomized controlled trials (parallel-group).

Exclusion

- Observational studies (cohort, case-control, cross-sectional).
- Case series or case reports.
- Cross-over trials.
- Simulation or volunteer studies.
- Conference abstracts without sufficient data for risk of bias assessment and effect estimation.

3.2 Information sources

The following databases will be searched from inception:

- MEDLINE (via PubMed)
- Embase (through Ovid)

Additional identification:

- Forward citation searching ("snowballing") of included trials
- Screening trial registries (ClinicalTrials.gov and WHO ICTRP) for contextual information on completed or ongoing trials

Only randomized trials will be included. Only studies published in English will be considered.

3.3 Search strategy

A draft MEDLINE search strategy is as follows and will be refined with information specialist input:

```
(
  "Intubation, Intratracheal"[Mesh] OR intubat*[tiab] OR "tracheal intubation"[tiab] OR
  "rapid sequence"[tiab] OR "rapid-sequence"[tiab] OR RSI[tiab]
)
AND
(
  "Emergency Service, Hospital"[Mesh] OR "Intensive Care Units"[Mesh] OR
  emergency[tiab] OR "emergency department"[tiab] OR ED[tiab] OR
  "critical illness"[Mesh] OR "critically ill"[tiab] OR ICU[tiab] OR
  "prehospital"[tiab]
)
```

```
AND
(
  etomidate[tiab] OR ketamine[tiab] OR propofol[tiab] OR
  amidate[tiab] OR diprivan[tiab] OR ketofol[tiab]
)
AND
(
  randomized controlled trial[pt] OR controlled clinical trial[pt] OR
  random*[tiab] OR trial[tiab] OR "clinical trial"[pt]
)
NOT
(
  animals[mh] NOT humans[mh]
)
```

The initial Embase search is:

```
1. exp endotracheal intubation/ or exp rapid sequence induction/
2. (intubat* or "tracheal intubation" or "rapid sequence" or "rapid-sequence" or RSI).ti,ab.
3. 1 or 2
4. exp emergency ward/ or exp intensive care unit/ or exp critical illness/
5. (emergency or "emergency department" or ED or "critically ill" or ICU or prehospital).ti,a
6. 4 or 5
7. (etomidate or ketamine or propofol or amidate or diprivan or ketofol).ti,ab.
8. exp etomidate/ or exp ketamine/ or exp propofol/
9. 7 or 8
10. 3 and 6 and 9
11. randomized controlled trial/ or controlled clinical trial/
12. (random* or trial or RCT).ti,ab.
13. 11 or 12
14. 10 and 13
15. (animal/ or nonhuman/) not human/
16. 14 not 15
```

Final search strategies for all databases will be reported in an appendix to the main manuscript.

3.4 Study selection

Search results will be imported into reference management software and then into a systematic review platform for de-duplication and screening. Two reviewers (or a person/machine combination with human verification) will independently screen titles and abstracts for potential eligibility. Full texts of potentially eligible reports will then be assessed independently using the predefined criteria.

Disagreements at any stage will be resolved through discussion or, if necessary, by consultation with a third reviewer. Reasons for exclusion at the full-text stage will be recorded. The study selection process will be summarized in a PRISMA flow diagram.

3.5 Data extraction

Two reviewers (or a person/machine combination with human verification) will independently extract data using a standardized, piloted form. Extracted information will include:

- Study characteristics: authors, year, country, setting (ED, ICU, prehospital), single versus multicenter, funding source.
- Population: inclusion and exclusion criteria, sample size, age, sex, primary diagnosis, baseline severity scores, baseline hemodynamics and vasopressor use.
- Interventions and comparators: induction agent, dose, timing, co-induction or adjunct agents, neuromuscular blocker type and dose, preoxygenation strategy, and RSI protocolization.
- Outcomes: definitions, time windows, and arm-level results for all eligible endpoints.
- Risk of bias domains (see below).

No contact with study authors is planned. If multiple reports refer to the same trial, they will be collated and treated as a single study, using the most complete and recent data.

3.6 Risk of bias assessment

Two reviewers (with optional machine assistance and full human verification) will independently assess risk of bias using the Cochrane Risk of Bias 2 (RoB 2) tool, considering:

- Bias arising from the randomization process.
- Bias due to deviations from intended interventions.
- Bias due to missing outcome data.
- Bias in measurement of the outcome.
- Bias in selection of the reported result.

Judgements (low risk, some concerns, high risk) will be incorporated into sensitivity and subgroup analyses.

3.7 Effect measures

For binary outcomes (mortality, hypotension, cardiovascular collapse, vasopressor initiation, first-pass success), odds ratios with 95% CIs will be calculated from arm-level data. Where necessary, alternative effect measures will be converted to ORs using standard methods.

Continuous outcomes, if analyzed descriptively (for example, minimum mean arterial pressure), will be summarized using mean differences or standardized mean differences with 95% CIs. Continuous outcomes will not be the focus of the NMA.

3.8 Data synthesis

3.8.1 Pairwise meta-analysis

Where there are at least two sufficiently similar trials, conventional pairwise meta-analyses will be performed using random-effects models. Statistical heterogeneity will be quantified using τ^2 and I^2, and 95% prediction intervals will be presented where appropriate.

3.8.2 Network meta-analysis

A random-effects NMA in a frequentist framework (for example, using the `netmeta` package in R) will be used to estimate relative treatment effects between all pairs of eligible induction agents for each outcome.

The NMA will assume transitivity; this assumption will be evaluated by comparing potential effect modifiers—such as setting, baseline hemodynamics, and co-interventions—across treatment comparisons. Consistency between direct and indirect evidence will be assessed using:

- A global design-by-treatment interaction model.
- Local node-splitting or analogous approaches to compare direct and indirect estimates.

Treatment ranking will be summarized using P-scores or surface under the cumulative ranking (SUCRA) values, with appropriate caution regarding uncertainty and evidence quality.

3.9 Heterogeneity and inconsistency

Heterogeneity in pairwise meta-analyses will be assessed using τ^2 and I^2. In NMA, the between-study variance for each outcome will be estimated and explored through subgroup and sensitivity analyses when data permit.

Predefined potential effect modifiers include:

- Clinical setting (ED versus ICU versus prehospital).
- Baseline vasopressor use.
- Degree of RSI protocolization (strict protocol versus pragmatic practice).
- Overall risk of bias.

3.10 Subgroup and sensitivity analyses

Planned subgroup analyses include:

- Setting (ED, ICU, prehospital).
- Presence versus absence of vasopressor use at baseline.
- High versus low risk of bias trials.

Sensitivity analyses will consider:

- Excluding high risk of bias trials.
- Excluding small trials (for example, fewer than 30 participants per arm).
- Collapsing or removing ketofol as a separate node to assess its impact on network geometry and estimates.

3.11 Reporting bias assessment

Risk of bias due to missing results will be assessed by evaluating selective non-reporting of outcomes and, when there are at least ten studies for an outcome, by inspecting funnel plots and considering statistical tests for small-study effects. No author contact is planned to obtain missing data.

3.12 Certainty of evidence

The certainty of the evidence for key comparisons and outcomes will be assessed using the GRADE framework adapted for NMA (for example, using CINeMA), taking into account risk of bias, inconsistency, indirectness, imprecision, and publication bias. Summary-of-findings tables will present results for the most clinically relevant comparisons (for example, etomidate versus ketamine, ketamine versus propofol, etomidate versus propofol).

3.13 Ethics and dissemination

As this review uses only published aggregate data, formal ethics approval is not required. Findings will be disseminated through peer-reviewed publications and scientific meetings. Data and analysis code will be made available in an appropriate public repository, subject to journal policies.

Funding and conflicts of interest

The review has no external funding. Institutional support is provided by participating authors' institutions. Any conflicts of interest will be fully disclosed in the final manuscript.

References

Jonathan D. Casey, Kevin P. Seitz, Brian E. Driver, Kevin W. Gibbs, Adit A. Ginde, Stacy A. Trent, Derek W. Russell, Amelia L. Muhs, Matthew E. Prekker, John P. Gaillard, L. Jane Stewart, et al. Ketamine or etomidate for tracheal intubation of critically ill adults. *New England Journal of Medicine*, 2025. doi: 10.1056/NEJMoa2511420. Advance online publication.

Raysa Cristina Schmidt, Fernando Godinho Zampieri, Fernando Jose da Silva Ramos, Felipe Santos Cavatoni Serra, Lucas Petri Damiani, Flávio Geraldo Rezende de Freitas, and Flávia Ribeiro Machado. Prospective, randomized, controlled trial comparing propofol versus ketamine in rapid sequence intubation in critically ill patients (promine): protocol paper and statistical analysis plan. *Critical Care Science*, 37:e20250133, 2025. doi: 10.62675/2965-2774.20250133.
